# Relationship between operon preference and functional properties of persistent genes in bacterial genomes

**DOI:** 10.1186/1471-2164-11-71

**Published:** 2010-01-28

**Authors:** Marit S Bratlie, Jostein Johansen, Finn Drabløs

**Affiliations:** 1 Department of Cancer Research and Molecular Medicine, Norwegian University of Science and Technology, N-7006 Trondheim, Norway

## Abstract

**Background:**

Genes in bacteria may be organised into operons, leading to strict co-expression of the genes that participate in the same operon. However, comparisons between different bacterial genomes have shown that much of the operon structure is dynamic on an evolutionary time scale. This indicates that there are opposing effects influencing the tendency for operon formation, and these effects may be reflected in properties like evolutionary rate, complex formation, metabolic pathways and gene fusion.

**Results:**

We have used multi-species protein-protein comparisons to generate a high-quality set of genes that are persistent in bacterial genomes (i.e. they have close to universal distribution). We have analysed these genes with respect to operon participation and important functional properties, including evolutionary rate and protein-protein interactions.

**Conclusions:**

Genes for ribosomal proteins show a very slow rate of evolution. This is consistent with a strong tendency for the genes to participate in operons and for their proteins to be involved in essential and well defined complexes. Persistent genes for non-ribosomal proteins can be separated into two classes according to tendency to participate in operons. Those with a strong tendency for operon participation make proteins with fewer interaction partners that seem to participate in relatively static complexes and possibly linear pathways. Genes with a weak tendency for operon participation tend to produce proteins with more interaction partners, but possibly in more dynamic complexes and convergent pathways. Genes that are not regulated through operons are therefore more evolutionary constrained than the corresponding operon-associated genes and will on average evolve more slowly.

## Background

The purpose of this study has been to improve our understanding of operons in bacterial genomes by analysing the organisation of genes across a large set of genomes. Operons are considered to be one of the major structural and regulatory features of prokaryotic genomes [1], but our understanding of the driving forces behind operon formation and the balance between individually regulated genes versus genes in operons is still incomplete. The analyses performed in this study focus on the properties of genes and gene products in relationship to operon organisation.

Gene transcription is strongly regulated, and the transcription of individual genes is controlled by transcription factors. In prokaryotes the transcription of several genes can be coordinated by the organisation of these genes into operons, and normally about 50% of the genes in prokaryotes are found in operons [2]. An operon is transcribed into a single polycistronic mRNA, and the genes in an operon often (but not always) code for gene products in the same functional pathway.

The operon consists of a promoter, an operator site and a set of structural genes [3]. The RNA polymerase binds to the promoter site and initiates the transcription. The operon may contain a regulator gene, but this can also be situated elsewhere in the genome. The regulatory protein coded by the regulator gene can bind to the operator. A repressor will inhibit transcription of the structural genes whereas an inducer will bind to the repressor and prevent it from interacting with the operator [1,3]. The operon structure leads to a strongly coordinated expression or repression of a set of genes.

Genes in the same operon are usually separated by fewer than 20 intergenic base pairs and are often conserved across species by vertical inheritance. However, few operons remain intact over long periods of time and operon dispersal is evident in many genomes. The selfish operon hypothesis is one of several theories behind operon formation. This model implies that horizontal gene transfer plays a critical role in gene cluster and operon formation [4,5]. Others explain operon formation by the fact that new operons reduce the amount of regulatory information that is required to specify optimal expression patterns and therefore operons should be likely to evolve when the regulation is complex [6]. It has also been suggested that genes encoding subunits of a complex will benefit from being in an operon, because the stochastic differences between the protein levels are reduced [7]. The formation and folding of protein complexes may also occur more rapidly when the genes are co-located in an operon [8].

For most organisms knowledge of operon structure is based on computational methods. The most common operon prediction methods are using one or more of the following criteria: intergenic distance, conserved gene clusters, functional relation, sequence elements and experimental evidence [9,10]. We have used the operon prediction data from Janga et al. [11] in our analyses. These are signature-based predictions; regions upstream of first transcribed genes contain higher densities of sigma-70 promoter-like signals that distinguish them from regions upstream of genes in the middle of operons [11].

The investigation of properties related to operon structure and genome evolution builds on several previous studies that have identified orthologous gene sets and used these to study evolution [12-15]. Here we have used the continuously growing number of sequenced bacterial genomes to build a large but robust set of orthologous genes, allowing us to focus the analysis on genes that seem to be essential to bacterial survival in general, and not limited to specific classes of bacteria.

Orthologs are defined as genes derived from a single ancestral gene in the last common ancestor of the compared species [16] and normally they perform equivalent functions in all species. Orthologs can be divided into two subgroups; with and without paralogs. Paralogs are genes that are related via duplication events, and they may perform biologically distinct functions compared to their ancestral genes. Orthologs that are found only once in each genome are also known as singletons, whereas orthologs with paralogs are known as duplicates [17]. Sequence comparison between singletons and duplicates has for example been used to elucidate the relationship between gene duplication and evolutionary rate [17,18].

Orthologs that are found in most species are often identified as important and possibly essential genes. Several studies [19,13,14,20] have tried to identify essential genes by deriving a minimal set of genes needed to sustain a functioning cell under ideal conditions, normally meaning unlimited amounts of nutrients and no competition from other cells. The first two completely sequenced bacteria were *Haemophilus influenza *and *Mycoplasma genitalium*. With these two bacteria the first minimal gene set was produced. Both *H. influenza *and *M. genitalium *are parasitic bacteria with small genomes, and a comparison resulted in a minimal gene set consisting of 256 genes [20]. Parasitic bacteria may have extra genes for interaction with their host, thus some of the genes described in [20] as essential are actually not required for general survival and therefore not found in all organisms [15].

Several computer programs and strategies are available for identifying orthologs. Relevant examples are COGs [21], TribeMCL [22], InParanoid [23] and OrthoMCL [24]. The database of Clusters of Orthologous Groups (COGs) of proteins [21,25] is a phylogenetic classification of proteins encoded in bacteria, archaea and eukaryotes. It is based on an all-against-all Blast search of complete proteomes. The COGs are made with the assumption that any group of at least three proteins from distant genomes that are more similar to each other than to any other proteins from the same genomes are likely to belong to an orthologous gene family. This orthology concept is used to classify proteins into groups. The InParanoid algorithm is based on pair-wise similarity scores calculated by Blast and uses reciprocal best hits between two species to find orthologs. While InParanoid is able to handle only two genomes at a time, OrthoMCL is designed to work with multiple genomes. OrthoMCL also identifies inparalogs (genes duplicated subsequent to speciation [16]) to be included in orthologous groups as within-species Blast hits that are reciprocally better than between-species hits [24]. It has been shown that OrthoMCL has a very good overall performance, compared to several other orthology detection strategies [26].

*Escherichia coli *is one of the most studied model organisms by biologists, and the genome has been studied intensively over several decades. This bacterium can be grown easily in a simple nutrient broth in a culture bottle, and more molecular information is known than for any other living organism [27]. We have therefore chosen to use *E. coli *O157:H7 EDL933 as a reference organism in this study; the gene names are according to the gene names in *E. coli *and the results from the analyses are presented in the context of the *E. coli *genome. However, *E. coli *is here used only as a template for presenting the general results. This means that specific statements that are correct for the analysis in general may not be correct for *E. coli*, although they are presented in that context; e.g. a gene identified as an "operon gene" because it is integrated in an operon in a majority of the genomes may still be found as a non-operon gene in *E. coli*.

In this study we have used Blast and OrthoMCL to identify inter-genomic clusters of orthologous genes, followed by COG to verify and supplement the results obtained from OrthoMCL. We have focused on identifying orthologs that are found in nearly all bacterial genomes included in this study, in total 113 genomes. We have then used this gene set to analyse selected features related to gene properties, organisation and evolution. In particular we have studied the operon organisation of the relevant genomes, trying to elucidate important characteristics of genes with strong preference for operon organisation compared to more flexible genes.

## Results

### Introduction

First the vocabulary is briefly described. A gene is classified as *persistent *if it is found in more than 90% of the organisms examined. It has been shown that gene persistence is strongly correlated with essentiality [28]. Most of the persistent genes are therefore likely to be *essential*, but not necessarily under the specific experimental conditions used for testing essentiality. An *ortholog cluster *is a set of orthologous genes from different genomes, as identified by OrthoMCL, whereas a *gene cluster *is a set of neighbouring genes in the genome, organised e.g. in an operon. Each individual gene in an ortholog cluster may be part of an operon (*operon gene*) or not (*non-operon gene*) in a given genome. The ortholog cluster itself may be classified as having a strong or weak operon preference, depending on the fraction of genes in the cluster that are part of an operon. We will use the terms *strong *and *weak operon genes *to describe this. The proteins produced from these genes are described in the same way, as *strong *and *weak operon proteins*. The ortholog clusters are also classified as *duplicates *or *singletons*, depending on whether the cluster contains paralogs or not. A cluster is also classified as a singleton cluster if the paralogous gene is more than 80% identical to the original gene, as it is likely that the duplication has happened quite recently and that the copy potentially may be lost again. Some ortholog clusters are also classified as *fused *or *mixed*. In the "mixed" category 10% - 50% of the proteins in the cluster consist of fused domains, while in the "fused" category over 50% of the proteins are fused. The fused and mixed clusters where normally excluded from the statistical analysis (see later). The ribosomal proteins (*r-proteins*) were often analysed as a separate class, in accordance with previous studies (see e.g. [29]).

### Selection of bacterial genomes

From the initial genome set, consisting of all bacterial genomes that were fully sequenced at the time of the initial analysis, only the strain with the longest genome was kept, thereby reducing the risk for removing relevant genes from the analysis. Any additional genes found in that strain will only affect the analysis if they are present in more than 90% of all included genomes, and in that case it seems reasonable to classify them as persistent. This approach gave a total of 113 bacterial genomes, with 109 circular and 4 linear genomes. A total of 13 phyla are represented in the data set. The dominating phylum is Proteobacteria (63 genomes), followed by Firmicutes (17), Actinobacteria (9) and Cyanobacteria (7). The remaining phyla (Aquificae, Bacteroidetes/Cholorobi, Chlamydiae/Verrucomicrobia, Chloroflexi, Deinococcus-Thermus, Fusobacteria, Planctomycetes, Spirochaetes, Thermotogae) are represented with up to 4 genomes each. *Symbiobacterium thermophilum *has been classified both as an Actinobacterium (TIGR) [30] and as a Firmicutes (NCBI) [31]. In spite of the high G + C content in *S. thermophilum*, the genome is more similar to the Firmicutes, which consist preferably of low G + C content bacteria [32]. We chose to classify the bacterium as a Firmicutes. A full list of the bacteria that were used in the analysis is given in supplementary material ([Additional file 1: Supplemental Table S1]).

### Clustering of gene orthologs

A total of 367,271 protein sequences from the 113 bacterial genomes were used as input to Blast and OrthoMCL, which grouped 305,484 (83%) of these proteins into 27,295 clusters. The cluster size varied from 2 to 540 proteins, with a large number of clusters containing only 2 proteins. Amongst the clusters with more than 2 proteins a large group containing 113 proteins was observed. A graph showing cluster sizes is shown in supplementary material ([Additional file 1: Supplemental Figure S1]).

### Identification of persistent genes

40 clusters from the OrthoMCL output contained singletons found in all 113 organisms. That is, these clusters contained 113 proteins from 113 different species. In addition we included clusters containing genes from at least 90% of the genomes (i.e. 102 organisms) and clusters containing duplicates (paralogs). This resulted in a list of 248 clusters. For clusters with duplicates we identified the most likely ortholog in each case using a score system based on rank in the Blast E-value score list. In short, we assumed that real orthologs on average are more similar to other proteins in the same cluster than the corresponding paralogs. The real ortholog will therefore appear with a lower total rank based on sorted lists of E-values. This procedure is fully explained in Methods. There were 34 clusters with too similar rank scores for reliable identification of true orthologs. These clusters (*lolD, clpP, groEL, lysC, tkt, cdsA, rpmE, glyA, trxB, ddl, dnaJ, dapA, folD, tyrS, hit, rpe, adk, serS, corC, lgt, pldA, htrA, atpB, xerD, rnhB, pgi, accC, msbA, gap, tuf, lepB, yrdC, fusA *and *ssb*) represent persistent genes, but since errors in identification of orthologs may affect the analysis they were not included in the final data set. We also removed genes located on plasmids as they would have an undefined genomic distance in the analysis of gene clustering and gene order. By doing so one of the clusters (*recG*) was only found in 101 genomes and was therefore removed from our list. The final list contained 213 clusters (112 singletons and 101 duplicates). An overview of all the 213 clusters is given in the supplementary material ([Additional file 1: Supplemental Table S2]). This table shows cluster IDs in accordance with the output IDs from OrthoMCL and gene names from our chosen reference organism, *Escherichia coli *O157:H7 EDL933. The results are also compared to the COG database [21]. Not all proteins were initially classified into COGs, therefore we used COGnitor at NCBI [33] to classify the remaining proteins. The orthologous group classification in [Additional file 1: Supplemental Table S2] is based on the properties of the clustered proteins (singleton, duplicate, fused and mixed). As indicated in this table, we also find gene clusters with more than 113 genes in the singletons category. These are clusters which originally contained paralogs, but where removal of paralogous genes located on plasmids resulted in 113 genes. The distribution of functional categories of the 213 orthologous gene clusters is shown in Table 1.

**Table 1:** Distribution of functional classes

	COG functional class	Gene **distr**. (%)	Norm. gene distr. (%)	**COG distr**. (%)	Norm. COG distr. (%)
**J**	Translation, ribosomal structure and biogenesis	35.2	37.8	4.7	9.0

**L**	Replication, recombination and repair	12.6	13.5	4.6	8.8

**F**	Nucleotide transport and metabolism	8.3	8.9	1.8	3.5

**M**	Cell wall/membrane/envelope biogenesis	6.5	7.0	3.6	6.9

**O**	Posttranslational modification, protein turnover, chaperones	5.2	5.6	3.9	7.4

**R**	General function prediction only	4.8	-	13.6	-

**K**	Transcription	4.8	5.2	4.5	8.6

**H**	Coenzyme transport and metabolism	3.9	4.2	3.5	6.7

**U**	Intracellular trafficking, secretion and vesicular transport	3.5	3.8	3.1	6.0

**I**	Lipid transport and metabolism	2.6	2.8	1.8	3.5

**C**	Energy production and conversion	2.6	2.8	5.0	9.5

**S**	Function unknown	2.2	-	26.1	-

**D**	Cell cycle control, cell division, chromosome partitioning	2.2	2.4	1.4	2.6

**E**	Amino acid transport and metabolism	2.2	2.4	5.2	10.0

**G**	Carbohydrate transport and metabolism	1.7	1.8	4.5	8.6

**Q**	Secondary metabolites biosynthesis, transport and catabolism	1.3	1.4	1.7	3.2

**T**	Signal transduction mechanisms	0.4	0.4	2.9	5.6

Functional class distribution of the 213 genes found in our analysis compared to the general distribution in the full COG database (expressed as percentages). The normalised distribution is computed using only the COG groups actually found in the analysis, also excluding genes with only predicted or unknown function.

Most of the persistent genes that have been identified belong to the category of translation and replication, which is consistent with earlier studies [13,12]. This includes in particular a large group of r-proteins. The categories of translation, replication, nucleotide transport, posttranslational modification and cell wall processes are overrepresented in our gene set compared to both total and normalised gene distribution in the COG database. This trend is confirmed by analysis of statistical overrepresentation with DAVID [34,35], showing that gene ontology terms like translation, DNA replication, ribonucleotide binding, biopolymer modification and cell wall biogenesis are significantly overrepresented in the gene set when using *E. coli *as a reference (all p-values < 0.001 after Benjamini and Hochberg correction for multiple hypothesis testing). Similarly, genes involved in signal transduction mechanisms, carbohydrate transport, amino acid transport and energy production and conversion, as well as all categories not observed in the set of persistent genes, are underrepresented. Also, the category of predicted genes is underrepresented.

### Comparison to minimal bacterial gene sets

**Figure 1:**
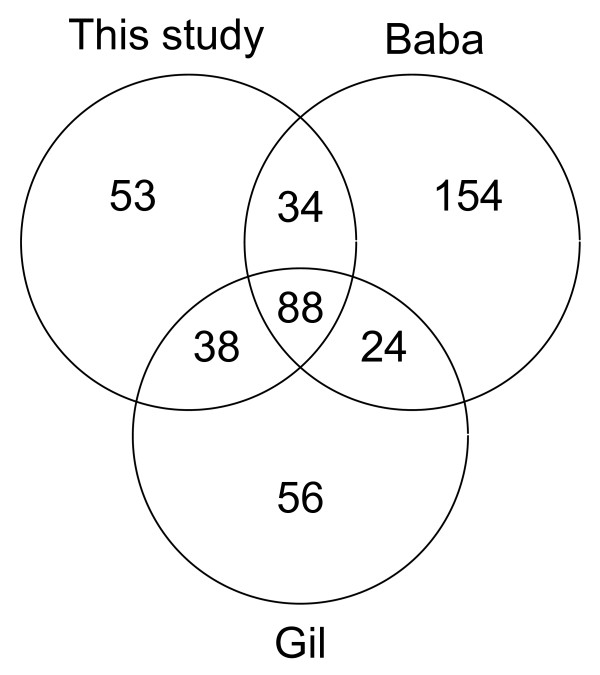
**Similarity to minimal gene sets**. Venn-diagram showing our gene set compared to the gene sets from Gil et al. [13] and Baba et al. [19]

We compared our list of 213 genes to various lists of essential genes for a minimal bacterium. Mushegian and Koonin [20] made a suggestion of a minimal gene set consisting of 256 genes, while Gil et al. [13] suggested a minimal set of 206 genes. Baba et al. [19] identified 303 possibly essential genes in *E. coli *by knockout studies (300 comparable). In a more recent paper of Glass et al. [14] a minimal gene set of 387 genes was suggested, whereas Charlebois and Doolittle [12] defined a core of all genes shared by sequenced genomes of prokaryotes (147 genomes; 130 bacteria and 17 archaea). This core consisted of 34 genes, including 11 r-proteins and 12 synthetases. Our core consists of 213 genes, including 45 r-proteins and 22 synthetases. Including archaea will result in a smaller core, and therefore our results are not directly comparable to the list from Charlebois and Doolittle [12]. By comparing our results to the gene lists from Gil et al. [13] and Baba et al. [19] we see a relatively good overlap (Figure 1). We have 53 genes in our list that are not included in the other gene sets ([Additional file 1: Supplemental Table S3]). As mentioned by Gil et al. [13] the largest category of conserved genes consists of those involved in protein synthesis, mainly aminoacyl-tRNA synthases and ribosomal proteins. As we see in Table 1 genes involved in translation represent the largest functional group in our gene set, contributing as much as 35%. One of the most important fundamental functions in all living cells is DNA replication, and this group constitutes about 13% of the total gene set in our data (Table 1).

### Genomic distribution of orthologs

**Figure 2:**
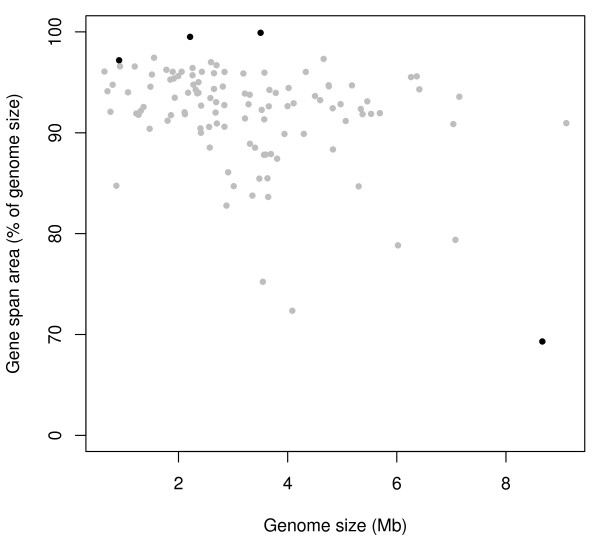
**Genomic range of persistent genes**. Genomic range of persistent genes expressed as a percentage of genome size. The four linear genomes are marked with black points; the grey points represent circular genomes.

**Figure 3:**
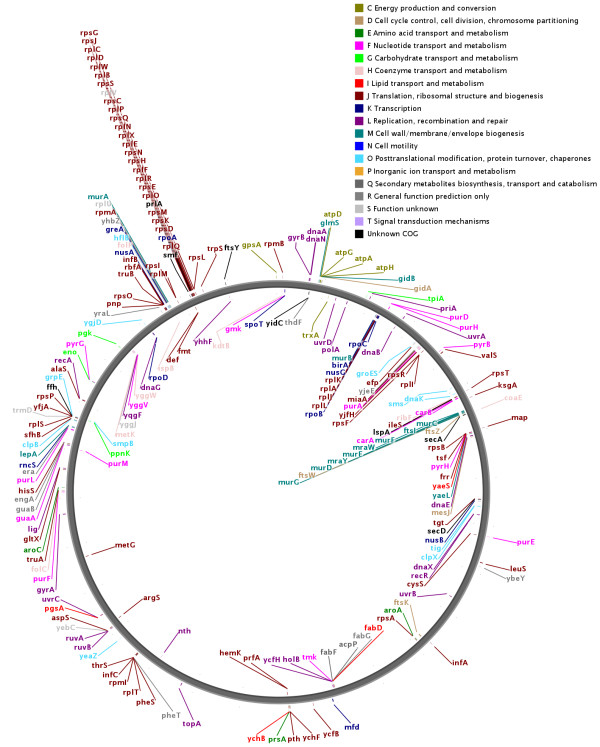
**Genomic distribution of persistent genes in *E. coli***. The genomic distribution of persistent genes shown as a genome atlas map for the reference organism *E. coli *O157:H7. The genes are colour coded according to COG group.

We looked into the possibility that gene clustering might be a large scale feature with clustering of persistent genes into specific genomic regions, rather than just a local operon-based feature. In Figure 2 the genomic range spanned by persistent genes is plotted for all the 113 genomes. In circular genomes the window giving the shortest range was always selected; the procedure is further explained in Methods. The values are calculated in percentage of genome size. As the figure shows, there is a clear tendency that the persistent genes in almost every case are spread throughout most (80-100%) of the genome. However, there are some bacteria where the persistent genes cover a slightly smaller genomic range (70-80%). These bacteria are *Geobacillus kaustophilus, Photobacterium profundum, Nocardia farcinica, Pseudomonas fluorescens *and *Streptomyces coelicolor*. It is important to point out that this analysis only shows that persistent genes tend to occupy a large genomic range. Most genes may still be clustered within that range. This is further analysed below, as part of the gene cluster and operon structure. Genomic distribution of the orthologs in *E. coli*, the chosen model organism, is shown as a genome atlas map [36] (Figure 3), and this confirms the global distribution of persistent genes.

We also looked into the possibility that specific gene pairs might show regular long range spacing across several genomes, for example due to large scale genome folding. This was done by comparing median distance (MED) to median absolute deviation (MAD) for all gene pairs. However, this did not reveal any long range regular spacing of persistent genes (data not shown).

**Figure 4:**
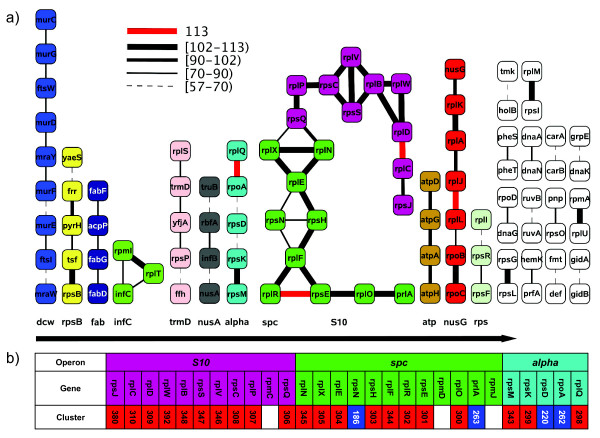
**Gene clusters from gene pair distances**. a) Gene clusters with intra-genomic pair-wise distance of at most 500 base pairs. Edges indicate the number of organisms where the distance is within this cut-off (see legend). b) An overview showing the persistent genes in the *S10, spc *and *alpha *operons found in *E. coli *O157:H7. The cluster number and gene type ([Additional file 1: Supplemental Table S2]; red: singletons, blue: duplicates) is also indicated.

We then looked at local gene clustering. We used Cytoscape [37] to visualise persistent gene pairs where at least 50% of the genomes had an intergenic distance of 500 base pairs or less (Figure 4a). This identified many relatively stable gene clusters. The dominating gene clusters correspond to well known operons, in particular the *str *locus containing the *S10*, *spc *and *alpha *operons. These operons constitute the longest array of genes conserved in bacterial genomes [38] and are found in almost all genomes. The *alpha *operon is separated from the *S10 *and *spc *operons. All of the genes in these three operons, except from *prlA *(*secY*) and *rpoA*, are genes coding for r-proteins. Figure 4b shows the genes involved in these three operons in *E. coli*. Also, the *atp *and *nusG/beta *clusters are strongly conserved, existing in most of the genomes. Other gene clusters in Figure 4a are the *dcw *(division and cell wall) cluster, the *rpsB *cluster, the *fab *cluster, the *infC *cluster, the *trmD *operon, the *nusA *cluster, and the *rps *cluster.

### Relative gene order of orthologs

**Figure 5:**
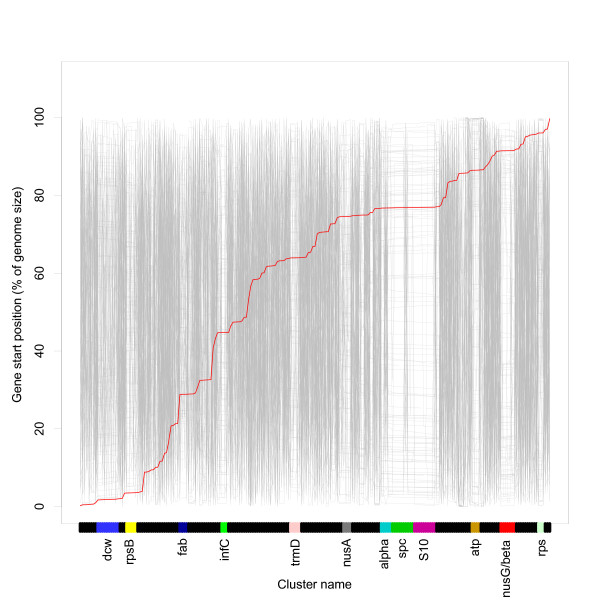
**Relative order of persistent genes in all genomes**. The red line indicates the gene order of the reference organism, *E. coli *O157:H7. For the other genomes the order of the persistent genes has been sorted according to the reference organism, and the relative genomic position of the genes plotted along the y-axis. Relatively flat horizontal lines in the plot indicate regions with conserved gene clustering compared to the reference organism (i.e. we are moving short genomic distances between genes when they are sorted according to the *E. coli *gene order). We see several such regions, marked with the same colours as in Figure 4. However, outside these regions the intra-genomic gene distances are highly variable.

We then looked into whether gene order of orthologous genes is conserved across genomes. The reference gene order and distribution in *E. coli *is shown in Figure 5. Also shown is the other organisms' gene distribution sorted according to the reference gene order. Relatively straight horizontal lines indicate compact gene clusters (short distance) with conserved gene order. A useful analogy may be a multiple sequence alignment: a sequence alignment aligns equivalent residues in different sequences and highlights regions with similar (conserved) residues, whereas the genome alignment in Figure 5 aligns equivalent genes in different genomes and highlights regions with similar (conserved) gene order. Gene clusters obtained from Figure 4a are indicated with the same colour in Figure 5.

Again we see a large region corresponding to the *alpha, spc *and *S10 *operons that is clearly conserved in most of the 113 organisms. This region is dominated by r-proteins, mostly singletons, and this conservation of gene order is likely to represent conserved operons. In general we see that gene clusters from cluster analysis (Figure 4) correlate very well with conserved regions in Figure 5.

**Figure 6:**
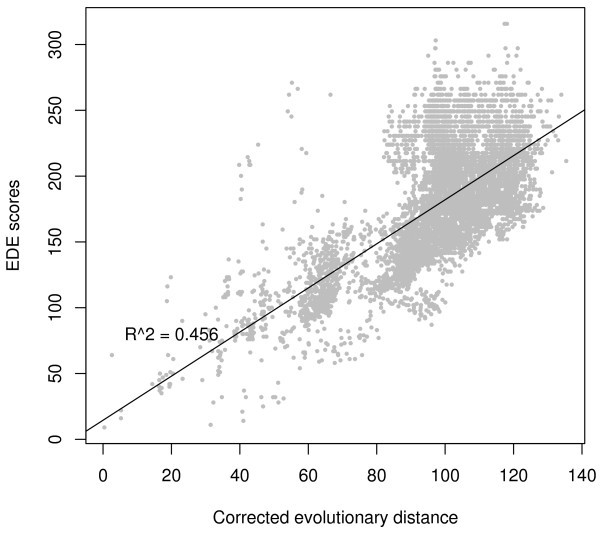
**Evolutionary distance between genomes**. Correlation between evolutionary distance from amino acid sequences for all persistent genes versus genomic gene order (EDE).

We then looked into whether variation in gene order observed in Figure 5 mainly reflects a normal evolutionary process, and therefore correlates with evolution in general. Distances between complete genomes can be computed by estimating the number of rearrangements needed to transform one genome into another based on gene order. Here we have used the Empirically Derived Estimator (EDE) approach [39]. By using the EDE corrected distances we got a measure of similarities in gene order between all 113 organisms. Additionally, evolution at the level of amino acid sequence was calculated from a multiple alignment of protein sequences of the persistent genes. Scoredist-corrected evolutionary distances [40] were calculated based on the BLOSUM62 matrix. Figure 6 plots distance by gene order (EDE score) compared to distance from amino acid sequence evolution. The figure shows that change in gene order is correlated with general sequence evolution, although the relationship is somewhat noisy.

**Figure 7:**
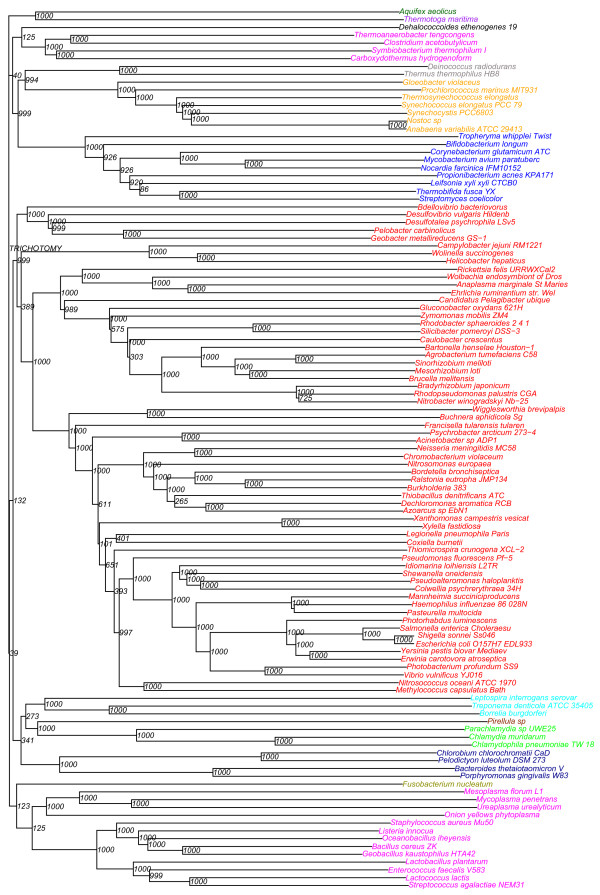
**Phylogram from persistent genes**. Phylogram based on a multiple alignment of protein sequences from the all persistent genes. Bacteria normally classified to the same phyla are marked with identical colour.

The general quality of the sequence set can to a certain extent be confirmed by a sequence-based phylogenetic analysis, compared to the known classification of the bacterial species. Figure 7 shows a phylogram computed on the combined multiple alignment of the persistent proteins, followed by a bootstrap analysis. A similar phylogenetic analysis was also done based on the EDE distances ([Additional file 1: Supplemental Figure S2]).

### Operon structure and properties

To analyse the actual operons we used the operon predictions from Janga et al. [11]. Only well defined singleton and duplicate clusters were used, i.e. not the fused (2 singletons, 3 duplicates) and mixed (1 singleton, 3 duplicates) clusters, giving a data set of 204 orthologs across 113 organisms.

We first investigated how often the individual genes were part of an operon. According to the above-mentioned operon predictions, the majority (76%) of our persistent genes take part in operons.

Next we tested whether operons show preference for singletons or duplicates. Counting the operon vs. non-operon distribution of the two different categories in the Janga predictions, we found that singletons are somewhat more often found in operons than duplicates ([Additional file 1: Supplemental Table S4], Fisher exact test odds ratio 1.19, p-value 3.725 × 10^-7^).

We then tested whether operons preferably consist of just one category (singletons or duplicates) or a mix of these two categories. By counting identical versus mixed gene pairs in the list by Janga et al. we found a clear tendency for identical pairs ([Additional file 1: Supplemental Table S5], odds ratio 1.28, p-value < 2.2 × 10^-16^). This probably reflects that it is more likely for the complete operon to be successfully duplicated rather than just one single gene.

The fraction of genes assigned to operon in each ortholog cluster was also related to COG categories. The results show that the average operon fraction varies from 67% in Posttranslational modification, protein turnover, chaperons (COG category O) to 85% in Cell wall/membrane/envelope biogenesis (COG category M) and Energy production and conversion (C) ([Additional file 1: Supplemental Table S6]).

For further analyses of operon structure we categorised all the 213 OrthoMCL gene clusters into strong and weak operon genes (also indicated in [Additional file 1: Supplemental Table S2]). A strong operon gene is defined as an OrthoMCL cluster where genes are located in an operon in at least 80% of the organisms, and this gave 110 strong and 103 weak operon genes. This gives a distinction between genes where operon organisation is essential versus genes where some regulatory flexibility is possible. This operon classification is given in [Additional file 1: Supplemental Table S2]. This set was further split into r-protein genes (45), strong operon genes (73) and weak operon genes (86), excluding fused and mixed genes as mentioned above, and this set of 204 genes was used for most of the following analyses.

**Figure 8:**
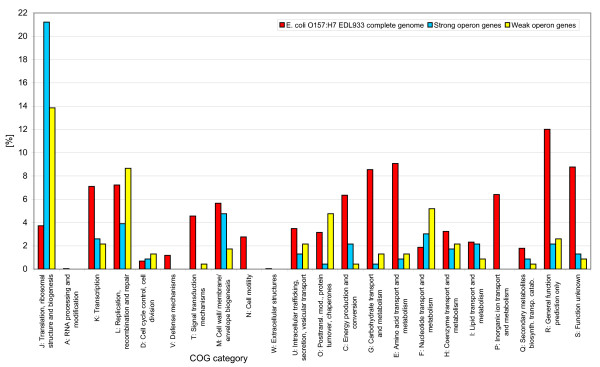
**Strong and weak operon genes according to COG categories**. The graph includes ribosomal genes (Translation, ribosomal structure and biogenesis (J)).

We looked at the distribution of strong and weak operon genes according to COG category and compared this to the overall distribution of COG categories in *E. coli *(Figure 8). Here r-protein genes were included. The strong operon genes are overrepresented in several of the COG categories compared to the weak operon genes; Translation, ribosomal structure and biogenesis (J), Transcription (K), Cell wall/membrane/envelope biogenesis (M), Energy production and conversion (C), Lipid transport and metabolism (I) and Secondary metabolites biosynthesis, transport and catabolism (Q). The translation category is strongly influenced by the r-protein genes. On the other hand, the weak operon genes are mainly overrepresented in Replication, recombination and repair (L), Posttranslational modification, protein turnover, chaperones (O) and Nucleotide transport and metabolism (F). This difference between strong and weak operon genes was confirmed with DAVID (excluding r-proteins), showing that whereas gene ontology terms like cell wall biogenesis and ATP metabolic process are overrepresented in strong operon genes, terms like DNA replication, response to stress and nucleotide binding are overrepresented in weak operon genes (p-values < 0.05 after Benjamini and Hochberg correction).

### Variation in evolutionary rate

In the phylogenetic analysis we looked at the total evolutionary distance based on all genes identified as persistent. However, there will obviously be inter-gene variation in the evolutionary rate. This was analysed by using pair-wise Blast bit scores normalised against alignment length; see Methods for further details.

#### Singleton versus duplicate genes

Earlier analyses [17] have found a difference in the evolutionary rate of singletons and duplicates, but this picture is strongly influenced by the 45 r-proteins in our data set. Analyses conducted with r-proteins included in the singletons category show that there is indeed a difference regarding the evolutionary rate. The median of the average bit scores (normalised over alignment length) is 0.81 for the singletons and 0.73 for the duplicates (data not shown), implying that genes in clusters dominated by singletons tend to be more similar to each other and evolve slower than duplicates. However, it is conventional to leave out r-proteins when looking at evolutionary rate [29] because they are highly expressed and evolve more slowly than other proteins. Without the r-proteins there was no significant difference between the singletons and duplicates (median of average bit scores 0.71 and 0.72 respectively). As expected the r-proteins evolve slowly with a median of average bit scores of 0.97. We also tested whether there was any difference regarding protein length for singletons and duplicates. When r-proteins were left out, this analysis did not give any significant difference.

#### Strong versus weak operon genes

**Figure 9:**
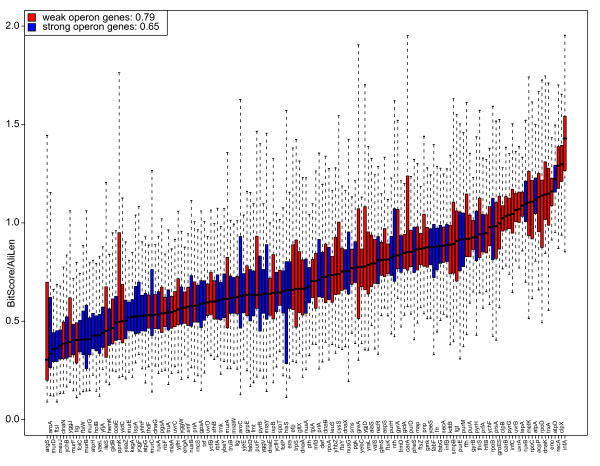
**Average protein bit score for strong and weak operon gene clusters**. A box plot showing the different gene clusters ranked according to average pair-wise bit score of the protein sequences (BitScore) normalised against alignment length (AliLen). The legend text shows the median score of each group (weak operon 0.79 bits, strong operon 0.65 bits). Ribosomal genes are not included. When they are included the numbers are 0.81 and 0.75, respectively.

**Figure 10:**
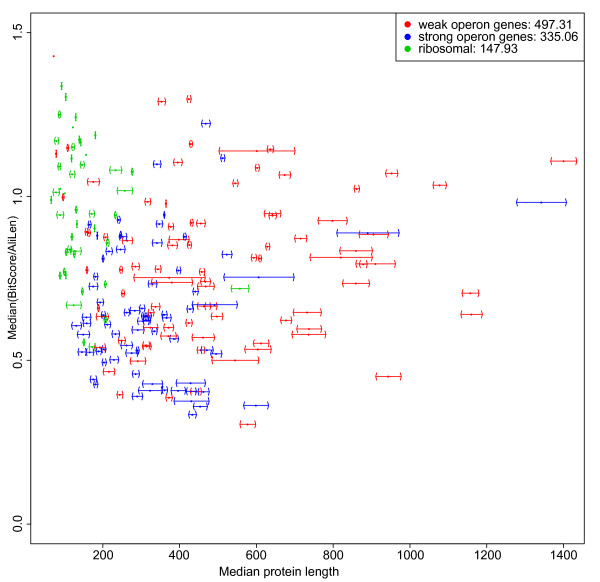
**Average protein length for strong and weak operon gene clusters**. The median protein sequence length over all 113 proteins for each of the 213 gene clusters plotted against median of normalised bit scores (see Figure 9). The legend text shows the median length for each group (weak operon 497.31 residues, strong operon 335.06 residues). This plot and analysis excludes ribosomal proteins; when they are included the corresponding number are 461.93 and 273.51, respectively.

We then performed the same analyses as described above, but comparing strong and weak operon proteins. The ribosomal and the fused/mixed proteins were left out of the analysis. The result is shown in Figure 9. The median of average bit scores for strong and weak operon proteins is 0.65 and 0.79 respectively, thus indicating that the strong operon genes evolve faster than the weak operon genes (p-value 3.527 × 10^-5^). As already mentioned the r-proteins have a median of average bit scores of 0.97. There is also a difference regarding protein length for strong and weak operon proteins. The proteins from weak operon genes (Figure 10) have an average length of 497.31 amino acids compared to 335.06 amino acids for proteins from strong operon genes (p-value 1.361 × 10^-5^).

### Protein-protein interactions

It has been proposed that evolutionary rate of a protein is influenced by the number of interaction partners [41]; all other things being equal a protein with more interactions should evolve more slowly. This is a controversial topic, as others [42] claim that slow evolutionary rate is a result of methods being biased towards counting more interactions for abundant proteins, and abundant proteins are known to evolve slowly.

We used the Molecular INTeraction Database (MINT) [43], and a reasonable subset of the 204 genes could be mapped to database entries (Table 2). We then counted interactions (both with and without self interactions) for the different groups of proteins (Table 2), and evaluated the significances by a bootstrap analysis with 10,000 permutations. The analysis showed significant overrepresentation of both strong and weak operon proteins in interaction with r-proteins and significant under-representation of direct contacts between strong and weak operon proteins and within weak operon proteins. It also showed that both strong and weak operon proteins have frequent self-interactions.

**Table 2:** Number of protein-protein interactions for the different classes.

Interaction		Ribosomal	Operon	Non-operon
		(45/43/43)	(73/45/29)	(86/62/52)
**Ribosomal**	MINT db	54 (57)	138	363
	Permutation	121.7 (120.1)	97.1 (111.9)	245.3 (242.3)
	P-value	1.0 (1.0)	0 (0)	0 (0)

**Operon**	MINT db		20 (62)	56
	Permutation		16.9 (26.0)	94.2 (112.8)
	P-value		0 (0)	1.0 (1.0)

**Non-operon**	MINT db			57 (110)
	Permutation			112.8 (121.7)
	P-value			1.0 (0.95)

The numbers shown are for all pairs of persistent genes found in MINT, both without (and with) self interactions included. The table also shows the average number of protein-protein interactions after 10,000 permutations, and estimated p-values for getting a number of interactions after permutation that is larger than the experimental one. The number of genes of each class in the full set, represented in MINT and found as interaction pairs in MINT is shown in the table header.

The 10 weak operon proteins that are responsible for the majority of the interactions with the r-proteins (i.e. they have more than 10 interactions) are in descending order InfC (with 35 interaction partners), SpoT, UvrC, PrsA, Pnp, DnaA, RpoC, Tgt, RpoD and Map (with 13 interaction partners).

In addition we counted the number of interactions for strong and weak operon proteins represented in the MINT database, and found on average 9.98 vs. 17.28 for strong vs. weak operon proteins, respectively, or 7.22 vs. 11.60 if we excluded r-proteins from the analysis.

### Interactions through shared pathways

In addition to direct physical interactions, genes and gene products may also interact indirectly through shared metabolic pathways and processes. This was analysed using the KEGG database [44]. A total of 134 of our 204 proteins could be mapped to KEGG, and 129 of these shared at least one pathway ([Additional file 1: Supplemental Table S7]). These proteins and pathways were then counted and analysed statistically, using a permutation test ([Additional file 1: Supplemental Table S7]). The analysis showed a statistically significant preference for strong operon proteins to share pathway (p-value 0.001), and a significant lack of preference for shared pathways between strong and weak operon genes (p-value 0.988). In addition we counted the number of pathways assigned to each protein for strong and weak operon proteins, and saw a general preference for weak operon proteins to be associated with more pathways than strong operon proteins ([Additional file 1: Supplemental Figure S3]).

## Discussion

### Identification of persistent orthologs

The first step in our analysis was to identify orthologous genes with close to universal distribution in a large set of bacterial genomes, as such genes are more likely to represent important functional and regulatory features that have been conserved during evolution. The set of publicly available genomes has a bias towards more extensively studied bacteria, and in order to reduce this bias somewhat we removed alternative strains of the same bacterium, in each case keeping the longest genome for analysis. This will obviously not remove bias completely, but it is a reasonable first step towards a less biased data set. We did not remove symbiotic bacteria from the analysis, although such bacteria may survive with a reduced genome by utilising host genes. The main reason for this decision was that we wanted to focus on genes that normally are kept under strict control within the target genome, and although the host may take over regulation of selected bacterial genes, this may not change the need for strict co-regulation of certain gene sets through an operon structure. However, we relaxed the inclusion criterion slightly by allowing any particular gene to be missing in up to 10% of the genomes.

We identified 213 persistent genes in total, based on the corresponding protein sequences ([Additional file 1: Supplemental Table S2]). This includes 69 genes found in all 113 organisms (61% from the COG Translation, ribosomal structure and biogenesis (J) category, in particular ribosomal genes), and 144 additional genes that could be found in at least 90% of the genomes.

There are several reasons why otherwise persistent genes may appear to be missing in individual genomes. It is possible that some of the missing genes actually were in the input data, but that they were not retrieved during the Blast search. It is also possible that legitimate orthologs were missed by OrthoMCL.

Some of the bacteria in our data set depend upon close parasitic or mutualistic relationship with eukaryotic hosts. This includes bacteria like *Mycoplasma penetrans, Onion yellows phytoplasma*, and *Wigglesworthia brevipalpis*. Mycoplasmas are among the smallest self-replicating organisms that are known today, and they have lost biochemical pathways such as amino acid and fatty acid biosynthesis [45]. Such bacteria exploit genes from the host, and may therefore have a smaller genome than free-living bacteria [12]. This can explain some missing genes. However, it has been shown that inclusion of organisms with reduced genomes improves the prediction of essentiality based on identification of persistent genes [28], which is an argument in favour of including such genomes in the analysis.

Non-orthologous gene displacement (NOD) is a mechanism by which essential genes may appear to be lost. In that case non-orthologous genes are coding for the same function in different bacteria [46]. These genes may be unrelated to each other, or they may be paralogs without significant similarity, and thus sequence similarity searches will not pick up these genes. This can be a possible explanation for some of the missing genes in our case, but we have not looked further into this.

During evolution proteins undergo different evolutionary processes, and some of these processes involve gene fusion, leading to more complex proteins. Thus groups of proteins may exist as individual genes in some organisms and as fused, multifunctional genes in other organisms. Previous analyses have shown that gene fusion occurs approximately four times more often than gene fission [47]. Multi-domain proteins or so-called fused proteins represent a challenge when doing protein clustering. A fused protein with two domains will fit into two different clusters, and the challenge is to find out which cluster to put it in. The most reasonable solution would be to put it into two different protein clusters, but this is not how OrthoMCL and many other tools will handle the situation. OrthoMCL clustering is based on Blast results, and when sorting proteins into clusters the program considers the E-values. If a protein consists of two fused domains, the domain with the best E-value will be used for cluster assignment. This results in a missing protein in another cluster, which of course is a problem if we are looking for globally persistent genes. In 9 out of the 213 clusters we found fused proteins.

The final gene set is dominated by ribosomal proteins. In *E. coli *53 r-proteins have been identified [48], but our analysis reveals that only 23 r-proteins are persistent in all of the 113 organisms. By also including r-proteins that are persistent according to our 90% cut off criterion we get a total of 45 r-proteins in our data set. The r-proteins are generally well conserved during evolution and are found in both prokaryotes and eukaryotes. In prokaryotes, the genes coding for r-proteins are often found in conserved operons [49]. Our results also show that the ubiquitous r-proteins are generally found with only one copy in the genome, as only 6 of the 45 persistent r-proteins are found with duplicates. The duplicated genes are *rpsD *(S4), *rpsN *(S14), *rpsQ *(S17), *rpsR *(S18) and *rpmB *(L28).

### Persistent orthologs represent essential genes

It is reasonable to expect that persistent orthologs are somehow essential to cell survival, as they by definition are well conserved across bacteria. This is a desired feature in this study, as it makes it more likely that regulatory or genomic structural features associated with these genes are conserved. It is relevant to compare this gene set to other essential gene sets, as this may serve as a quality check, and also indicate important differences between alternative strategies for identification of essential genes. Baba et al. [19] have identified 303 essential genes in *E. coli *K-12 by knockout experiments. Although our gene set is smaller than this list, the overlap is reasonably good; 122 of our 213 genes are essential according to these knockout experiments. There may be several reasons why the rest of the genes have not been identified as essential. In addition to potential experimental problems (incomplete knockout) there may be backup genes due to gene duplication (paralogs), or the genes may be essential only under non-laboratory conditions (e.g. stress handling). Gil et al. [13] used a consensus strategy integrating different types of information as well as previous minimal gene sets to define the core of a minimal gene set "able to sustain a functional bacterial cell under ideal conditions". When comparing our gene set to both Gil et al. [13] and Baba et al. [19] there are 53 genes that are unique to our gene set ([Additional file 1: Supplemental Table S3]). The dominating COG categories for these genes are Nucleotide transport and metabolism (F), Translation, ribosomal structure and biogenesis (J) and Replication, recombination and repair (L). Many of these genes are involved in processes that may become active during stress. Our gene list includes genes that encode heat shock proteins and proteins that induce the SOS response, for instance UvrA, UvrB and UvrC, which all are a part of the UvrABC nucleotide excision repair complex [50]. The UvrD protein is a helicase involved in DNA repair [51]. The Tig protein (also in our list) is together with DnaK involved in folding of newly synthesised proteins, and it has been shown that cells without *tig *and *dnaK *are not viable above 30°C [52]. We also find the genes *ruvA *and *ruvB *encoding proteins in the RuvABC complex. This complex functions in recombination pathways by binding to recombinational junctions and catalyzing strand cleavage. The *ruv *locus has been shown to be induced during the SOS response to DNA damage [53]. The *recA *and *recR *genes are also important in repairing DNA damage. Such genes, as well as others not described here, are essential for long-term survival under stress, but may not be identified as essential under controlled and non-stressful laboratory conditions.

Bubunenko et al. [54] have looked at the essentiality of ribosomal and transcription anti-termination proteins. According to their results, the majority of the 30S protein genes are essential, except the ribosomal protein genes *rpsF, rpsI, rpsM, rpsO, rpsQ *and *rpsT*. All of these last-mentioned genes are included in our list, and *rpsI*, *rpsM *and *rpsQ *were also listed as essential by Baba et al. [19] and Gil et al. [13].

Altogether, the gene set identified here seems to have more genes that are important for stress tolerance than some of the experimental gene sets. This is confirmed by testing our subset of 53 genes not found in Baba et al. or Gil et al. with statistical overrepresentation against the *E. coli *genome with DAVID. This shows that gene ontology terms like DNA repair, response to stress and SOS response are significantly overrepresented in this subset (p-values < 0.05 after Benjamini and Hochberg correction).

### The gene set can reproduce established bacterial classification

Extensive similarity of genes across bacterial species may in principle be the result of widespread horizontal gene transfer. Although this seems very unlikely for this data set, a phylogenetic analysis of the data may still help to indicate potential problems. The analysis in Figure 7 is based on merged protein sequences made from the 213 persistent genes. Not all of the genomes will contain all the 213 genes, as we included clusters found in at least 90% of the genomes. However, the resulting phylogram is still very robust with most bootstrap values close to 100%, except for a small number of branches (15) with bootstrap values below 50%. We see that the analysis is in excellent agreement with the known bacterial classification, and does not indicate any potential problems.

The correlation analysis of alignment distance vs. EDE distance shows a clear correlation (Figure 6). However, the phylogram based on EDE distances ([Additional file 1: Supplemental Figure S2]) is somewhat less consistent with known bacterial classification. It is difficult to say whether this is because gene order changes represent a more complex evolutionary process (i.e. less easily captured by a single distance measure), or whether it reflects real differences between these two processes.

### Persistent genes are distributed across the genomes

We see in Figure 2 that most genomes have the persistent genes spread throughout the whole genome, relatively independent of genome size. The genome with the smallest relative gene span is *S. coelicolor*, which is one of the largest genomes in our data set. *S. coelicolor *has a linear genome with a centrally located origin of replication [55], and it has the genes located in the middle of the chromosome. According to Bentley et al. [55] many streptomycetes can under laboratory conditions undergo deletions and insertions at either end of the chromosome without compromising viability. This can be a reasonable explanation for the organisation of persistent genes in *S. coelicolor*.

The genomic distribution in *E. coli *O157:H7 in Figure 3 confirms the results from Figure 2; we see that the persistent genes are distributed throughout most of the genome. Though we here see clear instances of clustering, much of this is likely to represent operons. In addition to the local clustering, there is a clear tendency for neighbouring clusters to be located on the same strand. There also seems to be some degree of clustering with respect to COG categories; relevant examples are Cell wall/membrane/envelope biogenesis (M) and Energy production and conversion (C). However, this has not been investigated in more detail.

### Operon structure is partly conserved

The initial operon analysis was based on a general clustering strategy with a quite relaxed criterion on gene distance. This should give a relatively unbiased overview, independent of any specific operon definition. This was then followed by a more operon-specific analysis, based on the operon data by Janga et al. [11].

In general the gene clusters identified by the cluster analysis represent known operons. If we compare Figure 4a, which is based on clustering on intra-genomic gene distances, and Figure 5, which is showing gene order conservation, we see that gene clusters representing operon-like structures are quite easy to recognise. In particular structures related to the *S10, spc *and *alpha *operons in *E. coli *are clearly visible. The *spc *operon is the most variable one, with 10 out of 12 genes persistent (*rpmD *and *rpmJ *are missing). In the *S10 *operon, only *rpmC *is missing due to our threshold (found in 100 genomes), whereas all genes of the *alpha *operon are found in all of the 113 organisms. We also see that these operons mainly consist of singletons (21 out of 25 genes). This underlines the evolutionary importance of these genes, as the lack of paralogs most likely means that they are under more strict control and selection than most other genes.

There are also other gene clusters that correspond to known operons. One of the largest clusters contains genes belonging to the division and cell wall (*dcw*) operon in *E. coli *[56], and contains *mur*, *fts *and *mra *genes. The genes *nusG-rplKAJL-rpoB *belong to the well-known *beta *operon, which is a classic bacterial gene cluster [57]. Four of the genes in the next cluster (*rpsP-yfjA-trmD-rplS*) are known to be a part of the *trmD *operon [58] in *E. coli*. *RplS*, *rpsP *and the flanking gene *ffh *are known to be essential for viability. Deletion of the *yfjA *gene results in a five-fold reduced growth rate of the cells [58]. The next cluster contains among others the genes *tsf/pyrH*, that are a part of the common cluster *tsf-pyrH-frr *[59]. The product of *pyrH *is involved in biosynthesis, while the products of *tsf *and *frr *are involved in translation. Janga et al. [59] suggest that the conservation might be accounted for by the general importance of macromolecular biosynthesis rather than from a direct functional relationship. We also see that the *metY-nusA-infB *operon is represented. This operon encodes functions involved in both transcription and translation [60], and the *nusA *gene is known to be involved in feedback control of the operon [61]. The cluster lacks the *metY, rpsO *and *pnp *genes. However, *rpsO *and *pnp *are found as a small separate cluster consisting of only two genes, as shown in Figure 4. The full gene order in this operon is therefore not sufficiently conserved among the 113 genomes to be identified.

The genes encoding several important fatty acid biosynthetic enzymes, the *fab *cluster, are found with the gene order *plsX-fabH-fabD-fabG-acpP-fabF *in *E. coli*, and several of these genes are known to be essential for growth [62]. We have identified 4 of these genes here. Genes representing an incomplete *atp *(or *unc*) operon are also found as a cluster in Figure 4a. The genes identified here are encoding the ATP synthase subunit B, C, A and D respectively. There are two additional clusters identified in our figure with a cluster size of three genes. One of the clusters is a purely ribosomal protein cluster, while the other one contains the *infC *gene encoding the translation initiation factor IF-3 in addition to two genes encoding ribosomal proteins.

There are several hypotheses regarding operon-formation, and the selfish-operon hypothesis [5] is maybe one of the most referred models. In this model, horizontal transfer of the complete operon is favoured over transfer of single genes, by natural selection. In this way, co-regulation and co-expression will still be conserved. At the time when the selfish-operon hypothesis was suggested, it was believed that genes providing for essential functions would not be part of an operon because these genes could not undergo the cycles of gene loss and gain that is one of the hallmarks of the model. However, it has later been found that essential genes preferentially are located in operons [63], and that essential and other ubiquitous genes still form new operons at significant rate [6]. According to a work done by Fang et al. [64] genes present in the majority of organisms and genes present in very few organisms have a strong clustering tendency, while genes of the intermediary category do not cluster.

From our work it is quite obvious that most of the persistent genes are found in operons. In particular the r-proteins stand out, as 35 of the 45 persistent r-proteins are found in operons in more than 80% of the genomes, and the genes encoding RplD, RplP, RplB, RplW, RpsK and RpsS are part of an operon in more than 110 of the genomes. Four of these proteins are part of the large ribosomal subunit, which may indicate that proper assembly of this subunit is particularly sensitive to a correct stoichiometric ratio for these proteins compared to other ribosomal components. For example r-protein RpsT is found as an operon gene in only 22% of the genomes, and the *rpsT *gene is not regarded as essential according to Bubunenko et al. [54].

From the statistics regarding operon structure in the different COG categories (based on predictions from Janga et al. [11]), the genes in Energy production and conversion (C) and Cell wall/membrane/envelope biogenesis (M) have the largest degree of operon organisation. Within these COG categories 85% of the genes in our data set are found to be a part of an operon. As already mentioned, the essential genes are preferentially found in operons, and even in Post-translational modification, protein turnover, chaperones (O), which has the lowest degree of operon structure, we find as many as 67% of the genes to be in an operon. However, in order to fully explain these preferences we also have to consider the results on protein-protein interactions and metabolic pathways (see following sections).

Conservation of gene order has been studied in a number of earlier reports [65-67]. The conclusion is that strict gene order conservation seems to be very rare. Prokaryotic genomes can be extensively reshuffled during evolution, and the frequency of rearrangement seems to be related to the phylogenetic distances [65]. This is consistent with our results shown in Figure 5. Conservation of gene order in evolutionary distant genomes seems to be due to operon organisation [59]. This is also is in accordance with our findings, as the gene clusters with conserved order in general are associated with known operons.

### Weak operon proteins have more interaction partners

Our analyses of protein-protein interactions in the MINT database showed that weak operon proteins on average have more interaction partners than strong operon proteins (17.3 vs. 10.0) and they also have more self-interactions than strong operon proteins. It also showed (Table 2) that weak operon proteins interact more frequently with ribosomal proteins than with strong operon proteins and other weak operon proteins. But even if we exclude r-proteins from the analysis the weak operon proteins have more interaction partners than the strong operon proteins (11.6 vs. 7.2). The preference for r-proteins over other proteins (including strong operon proteins) is even more striking as the majority of the r-proteins show a very strong operon preference. Most of the interactions with r-proteins are formed by proteins from 10 weak operon genes; *infC, spoT, uvrC, prsA, pnp, dnaA, rpoC, tgt, rpoD *and *map*. These genes mainly belong to the COG categories Translation, ribosomal structure and biogenesis (J), Transcription (K) and Replication, recombination and repair (L). The *infC *gene is a part of the *infC-rpmI-rplT *operon [68] of ribosomal subunits. The *spoT *gene is encoding one of the two proteins that control the bacterial response to stress, and this gene has been shown to be associated with the pre-50S ribosomal particle [69]. *UvrC *encodes for the UvrC protein, a component of the UvrABC nucleotide excision repair complex [70]. The *PrsA *gene encodes ribose-phosphate pyrophosphokinase which is important for cellular metabolism and found ubiquitously among all free-living organisms [71]. The *pnp *gene encodes polyribonucleotide nucleotidyltransferase, PNPase, in *E. coli*. PNPase is a component of the RNA degradosome complex [72]. DnaA, encoded by *dnaA*, initiates the chromosomal replication [73] while *rpoC *encodes the beta subunit of the RNA polymerase [74]. The *tgt *gene encodes the enzyme that is responsible for the posttranscriptional modification of specific tRNAs with queuine [75]. The *rpoD *gene encodes the RNA polymerase sigma 70 factor and is a part of an operon which also includes *rpsU *and *dnaG *[76]. The *map *(methionine aminopeptidase) gene has been found to be essential for cell growth in *E. coli *[77].

The number of interactions within the r-proteins is lower than anticipated. One source of error may be experimental bias in the MINT database. However, we do not think there is enough bias to seriously affect our analysis with respect to properties of strong vs. weak operon genes.

### Strong operon proteins are often in large complexes and linear pathways

Although interaction data often are related to shared pathways, these pathways may also include indirect interactions, e.g. through substrates. Therefore we also used data from the KEGG database in order to get a more complete picture of the interactome. From the KEGG database we retrieved how often ribosomal, strong and weak operon proteins were found in the same pathway ([Additional file 1: Supplemental Table S7]). This analysis is probably less reliable than the interaction analysis, as the concept of shared pathways is less clear cut than physical complex formation. However, we still believe that this analysis can indicate important properties associated with operon formation. All of the 45 ribosomal proteins were found in KEGG, while only 44 of the 73 strong operon proteins and 45 of the 86 weak operon proteins were found in the database. To be able to say how often the different groups are found in the same pathway, there was also a requirement that at least two genes from our data set had to be found in the same pathway, which further reduced the number of proteins to 41 and 43 for strong and weak operon proteins, respectively. The results showed that r-proteins were found only in pathways with other ribosomal proteins, while strong and weak operon proteins often were found in the same pathway. It also showed that weak operon proteins on average were found in more pathways than strong operon proteins. Whereas strong operon proteins mainly were assigned to just one pathway, weak operon proteins frequently were assigned to two or more pathways.

For further analysis we tried to categorise pathways with persistent genes into four different groups. The first group consists of large multi-protein complexes. Typical examples are r-proteins (KEGG ece03010) and the ATP synthetase complex (KEGG ece00190). In both cases the components are mainly strong operon proteins. An alternative route towards complex formation is a more step-wise process, where individual proteins are exchanged at each step. A relevant example is nucleotide excision repair (KEGG ece03420), with mainly weak operon proteins.

More traditional pathways can be subdivided in a similar way. A linear pathway consists of a series of steps in a relatively strict order. Typical examples are peptidoglycan synthesis (KEGG ece00550) and fatty acid biosynthesis (KEGG ece00061), in both cases dominated by strong operon proteins. The alternative layout consists of several relatively independent pathway steps linked to a common node, forming a convergent (or divergent) system. Examples are aminoacyl tRNA biosynthesis (KEGG ece00970), glutamate metabolism (KEGG ece00251) and valine, leucine and isoleucine biosynthesis (KEGG ece00400), in all cases dominated by weak operon proteins. However, several pathways do not show a clear preference for strong or weak operon proteins.

The preferences described above show that operons are beneficial mainly for large systems, where most components need to be available (in correct stoichiometric ratios) to make a functional system, like in relatively static multi-protein complexes or linear metabolic pathways. The operon structure is not equally essential when the interdependence between components is less strict, like in more dynamic complexes or convergent pathways. In these cases independent regulation may in fact be beneficial. This is also consistent with the observation that weak operon proteins tend to be used in more than one pathway, which may favour independent regulation.

### Weak operon genes evolve more slowly than strong operon genes

An important implication of the results described above is that weak operon genes should evolve more slowly than strong operon genes. Intuitively it may be tempting to assume the opposite, as operon formation may be associated with essential and tightly regulated genes under strong evolutionary selection. This is certainly the case for ribosomal proteins, where the rate of evolution is very low. However, this picture may change if we exclude the ribosomal proteins. As the weak operon proteins have more interaction partners and participate in more pathways, they may actually be more constrained with respect to evolution.

We sorted the strong and weak operon genes according to average protein alignment bit score of each gene cluster, excluding the r-proteins (Figure 9). This showed that weak operon genes on average evolve slower than strong operon genes. The result is to some extent dominated by genes at the extreme ends of the plot; a large group of fast evolving strong operon genes to the left and a similar group of slowly evolving weak operon genes to the right. The strong operon genes to the left are dominated by linear biosynthetic pathways, in particular peptidoglycan but also steroid, folat, pantothenate etc. The pattern to the right is less clear, but we find convergent pathways, chaperone subunits, DNA repair subunits and subunits for enzymatic complexes in metabolic pathways. This indicates that the rapidly evolving strong operon genes mainly are individual components of individual biosynthetic pathways that are relatively free to evolve, whereas proteins from the more slowly evolving weak operon genes to a larger extent have several partners (directly or indirectly), and therefore evolve more slowly.

We also used the same data to look for a similar bias for singleton genes compared to duplicates. A previous comparison of evolutionary rates in eukaryotes performed by Davis and Petrov [18] revealed a difference between duplicates and singletons. They examined the genomes of *S. cerevisiae *and *C. elegans*, and discovered that the duplicates in both genomes evolved slower than singletons. This observation is also supported by Jordan et al. [17], but they demonstrated that the evolutionary rate of duplicated genes accelerated immediately after duplication, before it subsequently slowed down. Our analysis did not reveal any significant differences. However, essential genes may be biased towards slow evolution independent of singleton/duplicate status. This may mask out the subtle trends that were observed in previous studies.

The analysis also showed that singletons are slightly overrepresented in strong operon genes. This basically shows that although these genes have more freedom to evolve through mutations, which only affects protein properties, they are less free to evolve through duplication, which will affect the actual gene regulation. This is consistent with the idea that operon genes in effect are more strongly regulated than non-operon genes.

This analysis may in principle be biased by the more rapid evolution in endosymbionts compared to free-living bacteria [78]. However, this will affect the result only if the *relative *rate of evolution between groups of genes is different for symbionts vs. non-symbionts, otherwise it will only affect the range of evolutionary rates that is observed.

### Weak operon genes are longer than strong operon genes

The above discussion shows that weak operon genes to a large extent represent independent subunits of otherwise complex systems. It is then relevant to ask whether this increased independence may have been achieved by integrating more functionality into each protein, e.g. by fusion of genes that normally form operons. In that case we should expect weak operon genes on average to be longer than operon genes.

The plot of median protein length for strong vs. weak operon genes showed a clear trend for strong operon genes to be on average shorter than weak operon genes (Figure 10). Gong et al. [79] have found that essential genes are overrepresented with respect to short and long genes, and underrepresented with respect to average length genes. However, this picture is not seen in our analysis. In *E. coli *the average protein length for the full proteome is 300 ± 231 amino acids (at 1 SD). In Figure 10 there seems to be relatively many proteins around 300 amino acids.

For large, essential complexes it may be a good strategy to assemble these from many small components that are easy to synthesise and fold rapidly (even under stress). Gong et al. argue that short genes have low cost (rapid synthesis, easy folding, no chaperones). This makes a relatively robust system, given that the genes are collected in an operon so that all components are produced simultaneously and in correct stoichiometric ratios. The same will be the case for well-defined linear biosynthetic pathways.

On the other hand, the increased length of weak operon genes indicates that these indeed may include multi-domain proteins, and although these may be more expensive to produce and fold correctly, the final products may be more robust and flexible with respect to how they may be used.

## Conclusions

This study illustrates the fundamental difference between ribosomal and non-ribosomal proteins in bacteria. Genes for ribosomal proteins have a strong tendency to participate in operons and the proteins are involved in formation of essential and well defined complexes, and this is consistent with a very slow rate of evolution. Persistent non-ribosomal proteins can be separated into two classes according to the tendency of the genes to participate in operons. Those with a strong tendency for operon participation have proteins with fewer interaction partners, but seem to participate in relatively static complexes and possibly linear pathways. Proteins from genes with a weak tendency for operon participation, on the other hand, tend to have more interaction partners, but possibly in more dynamic complexes and convergent pathways. This means that the proteins that are not regulated through operons actually are more evolutionary constrained than the corresponding operon-associated proteins and will therefore on average evolve more slowly.

## Methods

### BlastP and OrthoMCL

Complete proteomes from bacterial organisms were downloaded from NCBI ftp-server [80]. All-against-all analysis between full proteomes was done with BlastP [81] on a computer cluster with a total memory of 304 GB. Maximal number of Blast hits was set to 1000, unless the E-score from Blast exceeded a threshold of 10.

Results from BlastP were used as input to a modified version of OrthoMCL (version 1.4) [24] suitable for analysis on bacteria, and executed with default settings. OrthoMCL clustering data were loaded into a MySQL database (version 5.0) for further processing.

### Distinction between orthologs and paralogs

Identification of the most likely orthologous gene amongst duplicates was done by re-analysing Blast results for clusters with duplicated genes. It was assumed that true orthologs in general would be more similar to the other orthologs in the cluster, compared to the paralogs. This was assessed by comparing the ranking of gene copies in Blast output files for all non-duplicated genes in the cluster. The procedure is illustrated in [Additional file 1: Supplemental Figure S4] and described in detail in the supplementary material. The basic principle is that duplicated genes are assigned scores according to relative rank in Blast output files for non-duplicated genes from the same OrthoMCL cluster. The gene copy with lowest total rank score (i.e. largest tendency to appear first of the duplicated genes in the Blast output) is considered to be the most likely ortholog. A clear difference in total rank score between the first and the second gene copy shows that this gene copy is clearly more similar to the orthologs from other organisms in the cluster, and therefore more likely to be the true ortholog. We required the score difference to be at least 10% of the smallest possible rank score S_min _[Additional file 1] in order to make a reliable distinction between the ortholog and its paralogs, but in most cases the difference was significantly larger. If we do not consider horizontal gene transfer as a likely mechanism for these processes, this gene should be a reasonably good guess at the most likely ortholog. This seems to be supported by comparison with the essential genes identified by Baba et al. [19]. They have listed 11 cases where multiple genes have been found within the same COG class, indicating paralogs. For 6 cases where the list of homologs includes both essential and non-essential genes, according to knockout studies, our method selected the essential gene in 5 out of 6 cases. This is a reasonable result if we assume that orthologs are more likely to be essential than paralogs.

### Gene positions

Genes positioned on the lagging strand were reported with their start position subtracted from genome size. For linear genomes, the gene range was the difference in start position between the first and the last gene. For circular genomes we iterated over all possible neighbouring genes in each genome to find the longest possible distance. The shortest possible gene range was then found by subtracting the distance from the genome size. Thus, the shortest possible genomic range covered by persistent genes was always found.

### Data analysis

For data analysis in general, Python 2.4.2 was used to extract data from the database and the statistical scripting language R 2.5.0 was used for analysis and plotting. Gene pairs where at least 50% of the genomes had a distance of less than 500 bp were visualised using Cytoscape 2.6.0 [37]. The empirically derived estimator (EDE) [39] was used for calculating evolutionary distances from gene order, and the Scoredist corrected BLOSUM62 scores [40] were used for calculating evolutionary distances from protein sequences. ClustalW-MPI (version 0.13) [82] was used for multiple sequence alignment based on the 213 protein sequences, and these alignments were used for building a tree using the neighbour joining algorithm. The tree was bootstrapped 1000 times. The phylogram was plotted with the ape package developed for R [83].

Operon predictions were fetched from Janga et al. [11]. Fused and mixed clusters were excluded giving a data set of 204 orthologs across 113 organisms. We counted how often singletons and duplicates occurred in operons or not, and used the Fisher's exact test to check for significance.

Genes were further classified into strong and weak operon genes. If a gene was predicted to be in an operon in more than 80% of the organisms, the gene was classified as a strong operon gene. All other genes were classified as weak operon genes. Ribosomal proteins constituted a group by themselves.

Protein-protein interactions from the Molecular Interaction (MINT) database [43] were downloaded and 4852 interactions including genes from our list where extracted. Type of interactions across strong operon genes, weak operon genes and ribosomal genes were analysed and evaluated for significance by bootstrap analysis with 10,000 permutations on interactions.

Pathway data was downloaded from the KEGG database [44]. Data was organised in a list such that all possible gene pairs sharing the same pathway made an input to the list. Classification of strong operon, weak operon and ribosomal genes regarding pathway preference were analysed and bootstrapped for significance with 10,000 permutations.

Statistical overrepresentation of terms was analysed with DAVID [34,35], using p-values after Benjamini and Hochberg correction [84] for multiple hypothesis testing. The *E. coli *genome as represented in DAVID was used as reference for overrepresentation. DAVID computes overrepresentation for a large number of different terms. However, here we focussed on gene ontology (GO) [85] as a relatively unbiased term set.

## Authors' contributions

The project was initiated by FD. Data collection and analysis was done by MSB and JJ. MSB drafted the initial manuscript, and all authors contributed to the final version. All authors have read and approved the final manuscript.

## Supplementary Material

Additional file 1 Supplemental Tables S1 - S7 and Supplemental Figures S1 - S4. Click here for file
