# Supplementary material for: Relationship between operon preference and functional properties of persistent genes in bacterial genomes
Source: BMC Genomics. 2010 Jan 28;11:71. doi: 10.1186/1471-2164-11-71 (PMC2837039; doi:10.1186/1471-2164-11-71)
Supplement: Additional file 1 — Supplemental Tables S1 - S7 and Supplemental Figures S1 - S4. [file 1471-2164-11-71-S1.PDF]

Supplementary

Table S1 – The 113 organisms included in the study

Species	Phyla	Class
Bifidobacterium longum	Actinobacteria	Actinobacteria
Corynebacterium glutamicum ATCC 13032 Bielefeld	Actinobacteria	Actinobacteria
Leifsonia xyli xyli CTCB0	Actinobacteria	Actinobacteria
Mycobacterium avium paratuberculosis	Actinobacteria	Actinobacteria
Nocardia farcinica IFM10152	Actinobacteria	Actinobacteria
Propionibacterium acnes KPA171202	Actinobacteria	Actinobacteria
Streptomyces coelicolor	Actinobacteria	Actinobacteria
Thermobifida fusca YX	Actinobacteria	Actinobacteria
Tropheryma whipplei Twist	Actinobacteria	Actinobacteria
Aquifex aeolicus	Aquificae	Aquificae
Bacteroides thetaiotaomicron VPI.5482	Bacteroidetes/Chlorobi group	Bacteroidetes
Porphyromonas gingivalis W83	Bacteroidetes/Chlorobi group	Bacteroidetes
Chlorobium chlorochromatii CaD3	Bacteroidetes/Chlorobi group	Chlorobi
Pelodictyon luteolum DSM 273	Bacteroidetes/Chlorobi group	Chlorobi
Chlamydia muridarum	Chlamydiae/Verrucomicrobia group	Chlamydiae
Chlamydophila pneumoniae TW 183	Chlamydiae/Verrucomicrobia group	Chlamydiae
Parachlamydia sp UWE25	Chlamydiae/Verrucomicrobia group	Chlamydiae
Dehalococcoides ethenogenes 195	Chloroflexi	Dehalococcoidetes
Synechococcus elongatus PCC 7942	Cyanobacteria	Chroococcales
Synechocystis PCC6803	Cyanobacteria	Chroococcales
Thermosynechococcus elongatus	Cyanobacteria	Chroococcales
Gloeobacter violaceus	Cyanobacteria	Gloeobacteria
Anabaena variabilis ATCC 29413	Cyanobacteria	Nostocales
Nostoc sp	Cyanobacteria	Nostocales
Prochlorococcus marinus MIT9313	Cyanobacteria	Prochlorales
Deinococcus radiodurans	Deinococcus-Thermus	Deinococci
Thermus thermophilus HB8	Deinococcus-Thermus	Deinococci
Bacillus cereus ZK	Firmicutes	Bacilli
Enterococcus faecalis V583	Firmicutes	Bacilli
Geobacillus kaustophilus HTA426	Firmicutes	Bacilli
Lactobacillus plantarum	Firmicutes	Bacilli
Lactococcus lactis	Firmicutes	Bacilli
Listeria innocua	Firmicutes	Bacilli
Oceanobacillus iheyensis	Firmicutes	Bacilli
Staphylococcus aureus Mu50	Firmicutes	Bacilli
Streptococcus agalactiae NEM316	Firmicutes	Bacilli
Symbiobacterium thermophilum IAM14863	Firmicutes	Bacilli
Carboxydothermus hydrogenoformans Z.2901	Firmicutes	Clostridia
Clostridium acetobutylicum	Firmicutes	Clostridia
Thermoanaerobacter tengcongensis	Firmicutes	Clostridia
Mesoplasma florum L1	Firmicutes	Mollicutes

Mycoplasma penetrans	Firmicutes	Mollicutes
Onion yellows phytoplasma	Firmicutes	Mollicutes
Ureaplasma urealyticum	Firmicutes	Mollicutes
Fusobacterium nucleatum	Fusobacteria	Fusobacteria
Pirellula sp	Planctomycetes	Planctomycetacia
Agrobacterium tumefaciens C58 Cereon	Proteobacteria	Alphaproteobacteria
Anaplasma marginale St Maries	Proteobacteria	Alphaproteobacterie
Bartonella henselae Houston.1	Proteobacteria	Alphaproteobacterie
Bradyrhizobium japonicum	Proteobacteria	Alphaproteobacterie
Brucella melitensis	Proteobacteria	Alphaproteobacterie
Candidatus Pelagibacter ubique HTCC1062	Proteobacteria	Alphaproteobacteria
Caulobacter crescentus	Proteobacteria	Alphaproteobacteria
Ehrlichia ruminantium str. Welgevonden	Proteobacteria	Alphaproteobacteria
Gluconobacter oxydans 621H	Proteobacteria	Alphaproteobacteria
Mesorhizobium loti	Proteobacteria	Alphaproteobacteria
Nitrobacter winogradskyi Nb.255	Proteobacteria	Alphaproteobacteria
Rhodobacter sphaeroides 2 4 1	Proteobacteria	Alphaproteobacteria
Rickettsia felis URRWXCal2	Proteobacteria	Alphaproteobacteria
Silicibacter pomeroyi DSS.3	Proteobacteria	Alphaproteobacteria
Sinorhizobium meliloti	Proteobacteria	Alphaproteobacteria
Wolbachia endosymbiont of Drosophila melanogaster	Proteobacteria	Alphaproteobacteria
Zymomonas mobilis ZM4	Proteobacteria	Alphaproteobacteria
Azoarcus sp EbN1	Proteobacteria	Betaproteobacteria
Bordetella bronchiseptica	Proteobacteria	Betaproteobacteria
Burkholderia 383	Proteobacteria	Betaproteobacteria
Chromobacterium violaceum	Proteobacteria	Betaproteobacteria
Dechloromonas aromatica RCB	Proteobacteria	Betaproteobacteria
Neisseria meningitidis MC58	Proteobacteria	Betaproteobacteria
Nitrosomonas europaea	Proteobacteria	Betaproteobacteria
Ralstonia eutropha JMP134	Proteobacteria	Betaproteobacteria
Thiobacillus denitrificans ATCC 25259	Proteobacteria	Betaproteobacteria
Bdellovibrio bacteriovorus	Proteobacteria	delta/epsilon subdivisions
Campylobacter jejuni RM1221	Proteobacteria	delta/epsilon subdivisions
Desulfotalea psychrophila LSv54	Proteobacteria	delta/epsilon subdivisions
Desulfovibrio vulgaris Hildenborough	Proteobacteria	delta/epsilon subdivisions
Geobacter metallireducens GS.15	Proteobacteria	delta/epsilon subdivisions
Helicobacter hepaticus	Proteobacteria	delta/epsilon subdivisions
Pelobacter carbinolicus	Proteobacteria	delta/epsilon subdivisions
Wolinella succinogenes	Proteobacteria	delta/epsilon subdivisions
Acinetobacter sp ADP1	Proteobacteria	Gammaproteobacteria
Buchnera aphidicola Sg	Proteobacteria	Gammaproteobacteria
Colwellia psychrerythraea 34H	Proteobacteria	Gammaproteobacteria
Coxiella burnetii	Proteobacteria	Gammaproteobacteria
Erwinia carotovora atroseptica SCRI1043	Proteobacteria	Gammaproteobacteria
Escherichia coli O157H7 EDL933	Proteobacteria	Gammaproteobacteria
Francisella tularensis tularensis	Proteobacteria	Gammaproteobacteria
Haemophilus influenzae 86 028NP	Proteobacteria	Gammaproteobacteria
Idiomarina loihiensis L2TR	Proteobacteria	Gammaproteobacteria
Legionella pneumophila Paris	Proteobacteria	Gammaproteobacteria

Mannheimia succiniciproducens MBEL55E	Proteobacteria	Gammaproteobacteria
Methylococcus capsulatus Bath	Proteobacteria	Gammaproteobacteria
Nitrosococcus oceani ATCC 19707	Proteobacteria	Gammaproteobacteria
Pasteurella multocida	Proteobacteria	Gammaproteobacteria
Photobacterium profundum SS9	Proteobacteria	Gammaproteobacteria
Photorhabdus luminescens	Proteobacteria	Gammaproteobacteria
Pseudoalteromonas haloplanktis TAC125	Proteobacteria	Gammaproteobacteria
Pseudomonas fluorescens Pf.5	Proteobacteria	Gammaproteobacteria
Psychrobacter arcticum 273.4	Proteobacteria	Gammaproteobacteria
Rhodopseudomonas palustris CGA009	Proteobacteria	Gammaproteobacteria
Salmonella enterica Choleraesuis	Proteobacteria	Gammaproteobacteria
Shewanella oneidensis	Proteobacteria	Gammaproteobacteria
Shigella sonnei Ss046	Proteobacteria	Gammaproteobacteria
Thiomicrospira crunogena XCL.2	Proteobacteria	Gammaproteobacteria
Vibrio vulnificus YJ016	Proteobacteria	Gammaproteobacteria
Wigglesworthia brevipalpis	Proteobacteria	Gammaproteobacteria
Xanthomonas campestris vesicatoria 85.10	Proteobacteria	Gammaproteobacteria
Xylella fastidiosa	Proteobacteria	Gammaproteobacteria
Yersinia pestis biovar Mediaevails	Proteobacteria	Gammaproteobacteria
Borrelia burgdorferi	Spirochaetes	Spirochaetes
Leptospira interrogans serovar Lai	Spirochaetes	Spirochaetes
Treponema denticola ATCC 35405	Spirochaetes	Spirochaetes
Thermotoga maritima	Thermotogae	Thermotogae

Bacterial classification according to NCBI.

Table S2 – Persistent genes used for analysis

Nr	#	Gene name	Alt. name	COG	Cat	Op	Description
SINGLETONS							
240	116	*dnaX*		COG2812	L	1	DNA polymerase III subunits gamma and tau
248	115	*cysS*		COG0215	J	0	cysteinyl-tRNA synthetase
257	114	*rpsG*	*rpS7*	COG0049	Jr	1	30S ribosomal protein S7
258	114	*fmt*		COG0223	J	1	methionyl-tRNA formyltransferase
259	114	*yhbZ*	*obg*	COG0536	R	0	GTP-binding protein
275	113	*ksgA*		COG0030	J	1	dimethyladenosine transferase
276	113	*ychF*		COG0012	J	0	hypothetical protein/GTP-binding protein
277	113	*nusG*		COG0250	K	1	transcription antitermination protein NusG
278	113	*rpsI*	*rpS9*	COG0103*	Jr	1	30S ribosomal protein S9
279	113	*rplK*	*rpL11*	COG0080*	Jr	1	50S ribosomal protein L11
280	113	*rplA*	*rpL1*	COG0081*	Jr	1	50S ribosomal protein L1
281	113	*rpsL*	*rpS12*	COG0048*	Jr	0	30S ribosomal protein S12
282	113	*tsf*		COG0264*	J	1	elongation factor Ts
283	113	*frr*		COG0233*	J	1	ribosome recycling factor
284	113	*dnaG*		COG0358	L	0	DNA primase
285	113	*pheS*		COG0016	J	1	phenylalanyl-tRNA synthetase alpha subunit
287	113	*ygjD*	*gcp*	COG0533	O	0	O-sialoglycoprotein endopeptidase
288	113	*mraW*	*yabC*	COG0275*	M	1	S-adenosyl-methyltransferase
289	113	*leuS*		COG0495	J	0	leucyl-tRNA synthetases
290	113	*alaS*		COG0013	J	0	alanyl-tRNA synthetases
292	113	*tig*		COG0544	O	0	trigger factor
293	113	*infB*		COG0532	J	1	translation initiation factor IF-2
294	113	*nusA*		COG0195	K	1	transcription elongation factor NusA
296	113	*pyrG*		COG0504	F	0	CTP synthetases
297	113	*valS*		COG0525	J	0	valyl-tRNA synthetases
298	113	*rplQ*	*rpL17*	COG0203	Jr	1	50S ribosomal protein L17
299	113	*rpsK*	*rpS11*	COG0100	Jr	1	30S ribosomal protein S11
300	113	*rplO*	*rpL15*	COG0200	Jr	1	50S ribosomal protein L15
301	113	*rpsE*	*rpS5*	COG0098	Jr	1	30S ribosomal protein S5
302	113	*rplR*	*rpL18*	COG0256	Jr	1	50S ribosomal protein L18
303	113	*rpsH*	*rpS8*	COG0096*	Jr	1	30S ribosomal protein S8
304	113	*rplE*	*rpL5*	COG0094	Jr	1	50S ribosomal protein L5
305	113	*rplX*	*rpL24*	COG0198	Jr	1	50S ribosomal protein L24
307	113	*rplP*	*rpL16*	COG0197	Jr	1	50S ribosomal protein L16
308	113	*rpsC*	*rpS3*	COG0092	Jr	1	30S ribosomal protein S3
309	113	*rplD*	*rpL4*	COG0088*	Jr	1	50S ribosomal protein L4
310	113	*rplC*	*rpL3*	COG0087*	Jr	1	50S ribosomal protein L3
311	113	*rplT*	*rpL20*	COG0292*	Jr	0	50S ribosomal protein L20
312	113	*rpmA*	*rpL27*	COG0211*	Jr	1	50S ribosomal protein L27
314	113	*rpsO*	*rpS15*	COG0184*	Jr	0	30S ribosomal protein S15
315	113	*rpoC*		COG0086	K	0	DNA-directed RNA polymerase beta' subunit
317	113	*smpB*		COG0691	O	0	SsrA-binding protein
318	113	*rpsB*	*rpS2*	COG0052*	Jr	1	30S ribosomal protein S2
319	113	*rplL*	*rpL7/12*	COG0222	Jr	0	50S ribosomal protein L7/L12
320	113	*rplJ*	*rpL10*	COG0244	Jr	1	50S ribosomal protein L10
321	113	*prfA*		COG0216	J	1	peptide chain release factor 1

332	112	*rplM*	*rpL13*	COG0102*	Jr	1	50S ribosomal protein L13
333	112	*rpsP*	*rpS16*	COG0228*	Jr	1	30S ribosomal protein S16
334	112	*trmD*		COG4752	S	1	tRNA (guanine-N(1)-)-methyltransferase
335	112	*rplS*	*rpL19*	COG0335*	Jr	0	50S ribosomal protein L19
336	112	*rpsF*	*rpS6*	COG0360	Jr	1	30S ribosomal protein S6
337	112	*ybeY*		COG0319	R	1	hypothetical protein
339	112	*engA*		COG1160	R	0	GTP-binding protein EngA
342	112	*pgk*		COG0149	G	1	phosphoglycerate kinase
343	112	*rpsM*	*rpS13*	COG0099	Jr	1	30S ribosomal protein S13
344	112	*rplF*	*rpL6*	COG0097	Jr	1	50S ribosomal protein L6
345	112	*rplN*	*rpL14*	COG0093*	Jr	1	50S ribosomal protein L14
346	112	*rplV*	*rpL22*	COG4933	Sr	1	50S ribosomal protein L22
347	112	*rpsS*	*rpS19*	COG0185	Jr	1	30S ribosomal protein S19
348	112	*rplB*	*rpL2*	COG0090	Jr	1	50S ribosomal protein L2
349	112	*rplU*	*rpL21*	COG3743	Sr	1	50S ribosomal protein L21
351	112	*mesJ*		COG0037	D	1	hypothetical protein/cell cycle protein MesJ
358	111	*rplI*	*rpL9*	COG0359	Jr	1	50S ribosomal protein L9
360	111	*infC*		COG0290	J	0	translation initiation factor IF-3
361	111	*rpoB*		COG0085	K	1	DNA-directed RNA polymerase beta subunit
362	111	*ftsY*		COG0552	U	0	signal recognition particle-docking protein FtsY
363	111	*ffh*		COG0541	U	0	signal recognition particle protein
367	110	*coaE*		COG0237	H	1	dephospho-CoA kinase
371	110	*miaA*		COG0324	J	0	tRNA delta(2)-isopentenylpyrophosphate transferase
377	110	*pyrH*		COG0528	F	1	uridylate kinase
378	110	*uvrC*		COG0322	L	0	excinuclease ABC subunit C
380	110	*rpsJ*	*rpS10*	COG0051	Jr	1	30S ribosomal protein S10
381	110	*gmk*		COG0194	F	0	guanylate kinase
382	110	*uvrB*		COG0556	L	0	excinuclease ABC subunit B
383	109	*clpX*		COG1219	O	0	ATP-dependent protease ATP-binding subunit
385	109	*yhhF*	*rsmD*	COG0742	L	1	hypothetical protein/N6-adenine-specific methylase
388	109	*pnp*		COG1185	J	0	polyribonucleotide nucleotidyltransferase
389	109	*rpsA*	*rpS1*	COG0539	Jr	0	30S ribosomal protein S1
390	109	*priA*		COG1198	L	0	primosome assembly protein PriA
392	109	*rplW*	*rpL23*	COG0089	Jr	1	50S ribosomal protein L23
393	109	*sfhB*	*rluD*	COG0564	J	0	ribosomal large subunit pseudouridine synthase D
394	109	*rpsT*	*rpS20*	COG0268	Jr	0	30S ribosomal protein S20
412	108	*truB*		COG0130	J	1	tRNA pseudouridine synthase B
415	108	*tmk*		COG0125	F	1	thymidylate kinase
431	107	*ruvA*		COG0632	L	1	Holliday junction DNA helicase motor protein
432	107	*ruvB*		COG2255	L	1	Holliday junction DNA helicase RuvB
434	107	*nusB*		COG0781	K	1	transcription antitermination protein NusB
435	107	*rpmI*	*rpL35*	COG0291	Jr	0	50S ribosomal protein L35
440	107	*holB*		COG0470	L	1	DNA polymerase III subunit delta
444	106	*rbfA*		COG0858	J	1	ribosome-binding factor A
451	106	*kdtB*	*coaD*	COG0669	H	0	phosphopantetheine adenylyltransferase
456	106	*yjfH*	*rlmB*	COG0566	J	0	hypothetical protein/tRNA/rRNA methyltransferase
457	106	*recR*		COG0353	L	1	recombination protein RecR
464	105	*mfd*		COG1197	LK	0	transcription-repair coupling factor
466	105	*era*		COG1159	R	1	GTP-binding protein Era
468	105	*pgsA*		COG0558	I	0	CDP-diacylglycerol--glycerol-3-phosphate 3-phosphatidyltransferase

469	105	*yggW*	*hemN*	COG0635	H	1	coproporphyrinogen III oxidase
477	104	*aroC*		COG0082*	E	1	chorismate synthase
482	104	*mraY*		COG0472	M	1	phospho-N-acetylmuramoyl-pentapeptide-transferase
487	104	*ychB*	*ispE*	COG1947	I	1	4-diphosphocytidyl-2-C-methyl-D-erythritol kinase
489	104	*gidA*		COG0445	D	0	glucose-inhibited division protein A
490	103	*purM*		COG0150	F	1	phosphoribosylaminoimidazole synthetases
491	103	*sms*	*radA*	COG1066	O	0	probable ATP-dependent protease
492	103	*purD*		COG0151	F	0	phosphoribosylamine--glycine ligase
494	103	*yfjA*	*rimM*	COG0806	J	1	16S rRNA-processing protein
502	103	*yqgF*		COG0816*	L	1	Holliday junction resolvase-like protein
503	103	*thdF*	*mnmE*	COG0486	R	0	tRNA modification GTPase
511	102	*carA*		COG0505	EF	0	carbamoyl-phosphate synthase small subunit
517	102	*yeaZ*		COG1214	O	1	hypothetical protein/glycoprotease family protein
**FUSED SINGLETONS**							
416	108	*guaA*		COG0518<br>COG0519	F<br>F	0	bifunctional GMP synthase/glutamine amidotransferase protein
286	113	*pheT*		COG0072<br>COG0073	J<br>R	1	phenylalanyl-tRNA synthetase beta subunit
**MIXED SINGLETONS**							
501	103	*purL*		COG0046F	F	0	phosphoribosylformylglycinamidine synthase
**DUPLICATES**							
10	312	*fabG*		COG1028I	QR	1	3-ketoacyl-(acyl-carrier-protein) reductase
38	199	*clpB*		COG0542	O	0	ClpB protein
60	171	*uvrD*		COG0210	L	0	DNA helicase II
62	168	*fabF*		COG0304	IQ	1	3-oxoacyl-(acyl carrier protein) synthase
63	168	*rpoD*		COG0568	K	0	RNA polymerase sigma factor
82	160	*def*		COG0242*	J	1	peptide deformylase
99	149	*hflB*	*ftsH*	COG0465	O	0	cell division protein FtsH
101	148	*dnaE*		COG0587	L	0	DNA polymerase III subunit alpha
105	146	*map*		COG0024	J	0	methionine aminopeptidase
119	140	*spoT*	*relA*	COG0317	TK	0	GTP pyrophosphokinase
127	135	*yjeE*		COG0802	R	1	hypothetical protein
143	132	*uvrA*		COG0178	L	0	excinuclease ABC subunit A
145	131	*groES*		COG0234	O	0	co-chaperonin GroES
158	127	*dnaK*		COG0443	O	0	molecular chaperone DnaK
167	126	*dnaB*		COG0305	L	0	replicative DNA helicase
168	125	*murA*		COG0766	M	0	UDP-N-acetylglucosamine 1-carboxyvinyltransferase
173	124	*trxA*		COG0526	OC	0	thioredoxin
176	124	*infA*		COG0361	J	0	translation initiation factor IF-1
185	122	*ftsK*		COG1674	D	0	Cell division protein FtsK
186	122	*rpsN*	*rpS14*	COG0199	Jr	1	30S ribosomal protein S14
189	121	*ftsI*		COG0768	M	1	penicillin-binding protein
193	121	*trpS*		COG0180	J	0	tryptophanyl-tRNA synthetases
199	120	*yaeS*	*uppS*	COG0020	I	1	undecaprenyl pyrophosphate synthetases
202	120	*thrS*		COG0441	J	0	threonyl-tRNA synthetases
204	120	*yebC*		COG0217*	S	0	hypothetical protein
205	120	*gyrA*		COG0188	L	0	DNA gyrase subunit A
206	120	*dnaN*		COG0592	L	0	DNA polymerase III subunit beta
210	120	*ycfH*	*tatD*	COG0084	L	1	hypothetical protein/hydrolase TatD family
220	118	*rpsD*	*rpS4*	COG0522	Jr	0	30S ribosomal protein S4

221	118	*efp*		COG0231	J	0	elongation factor P
227	117	*prsA*		COG0462	FE	0	ribose-phosphate pyrophosphokinase
232	116	*gltX*		COG0008	J	0	glutamyl-tRNA synthetases
234	116	*ycfB*	*trmU*	COG0482	J	0	tRNA (5-methylaminomethyl-2-thiouridylate)-methyltransferase
237	116	*tpiA*		COG0149	G	0	triosephosphate isomerise
238	116	*secA*		COG0653	U	0	translocase
245	115	*rpsR*	*rpS18*	COG0238*	Jr	1	30S ribosomal protein S18
246	115	*ileS*		COG0060	J	0	isoleucyl-tRNA synthetases
247	115	*argS*		COG0018	J	0	arginyl-tRNA synthetases
249	115	*gyrB*		COG0187	L	0	DNA gyrase subunit B
250	115	*pth*		COG0193*	J	1	peptidyl-tRNA hydrolase
254	114	*lspA*		COG0597	MU	1	signal peptidase II
255	114	*glmS*		COG0449	M	0	D-fructose-6-phosphate amidotransferase
256	114	*hemK*		COG2890	J	1	modification methylase, HemK family
260	114	*grpE*		COG0576	O	0	grpE protein
261	114	*lepA*		COG0481	M	0	GTP-binding protein LepA
262	114	*rpoA*		COG0202	K	1	DNA-directed RNA polymerase alpha subunit
263	114	*prlA*	*secY*	COG0201	U	1	preprotein translocase SecY
265	114	*eno*		COG0148	G	0	phosphopyruvate hydratase
266	114	*lig*	*ligA*	COG0272	L	0	DNA ligase
267	114	*recA*		COG0468	L	0	recombinase A
269	113	*acpP*		COG0236	IQ	0	acyl carrier protein
271	113	*yidC*		COG0706	U	0	putative inner membrane protein translocase component YidC
273	113	*purA*		COG0104*	F	0	adenylosuccinate synthetases
295	113	*hisS*		COG0124	J	1	histidyl-tRNA synthetases
306	113	*rpsQ*	*rpS17*	COG0186	Jr	1	30S ribosomal protein S17
313	113	*rncS*		COG0571	K	1	ribonuclease III
322	113	*dnaA*		COG0593	L	0	chromosomal replication initiation protein
327	112	*ftsZ*		COG0206	D	0	cell division protein FtsZ
338	112	*metK*		COG0192	H	0	S-adenosylmethionine synthetases
340	112	*gpsA*		COG0240	C	1	NAD(P)H-dependent glycerol-3-phosphate dehydrogenase
341	112	*aspS*		COG0173	J	0	aspartyl-tRNA synthetases
356	111	*ftsW*		COG0772*	D	1	cell division protein FtsW
357	111	*murB*		COG0812	M	1	UDP-N-acetylenolpyruvoylglucosamine reductase
359	111	*ribF*		COG0196	H	0	hypothetical protein/riboflavin biosynthesis protein RibF
369	110	*carB*		COG0458	EF	0	carbamoyl-phosphate synthase large subunit
370	110	*murE*		COG0769	M	1	UDP-N-acetylmuramoylalanyl-D-glutamate--2,6-diaminopimelate ligase
373	110	*yaeL*	*ecfE*	COG0750	M	1	hypothetical protein/putative membrane-associated zinc metalloprotease
376	110	*truA*		COG0101	J	1	tRNA pseudouridine synthase A
379	110	*yggJ*	*rsmE*	COG1385	S	0	hypothetical protein
386	109	*fabD*		COG0331	I	1	acyl-carrier-protein S-malonyltransferase
387	109	*ispB*		COG0142	H	0	octaprenyl-diphosphate synthase
396	109	*rpmB*	*rpL28*	COG0227	Jr	0	50S ribosomal protein L28
403	108	*folC*		COG0285	H	1	folylpolyglutamate synthase
406	108	*nth*		COG0177	L	1	endonuclease III
409	108	*folP*		COG0294	H	1	7,8-dihydropteroate synthase
413	108	*ppnK*	*nadK*	COG0061	G	0	inorganic polyphosphate/ATP-NAD kinase
420	107	*aroA*		COG0128	E	1	3-phosphoshikimate 1-carboxyvinyltransferase
424	107	*murD*		COG0771	M	1	UDP-N-acetylmuramoyl-L-alanyl-D-glutamate synthetase
425	107	*murG*		COG0707	M	1	N-acetylglucosaminyl transferase

429	107	*pyrB*		COG0540	F	1	aspartate carbamoyltransferase catalytic subunit
433	107	*greA*		COG0782	K	0	transcription elongation factor GreA
436	107	*yraL*		COG0313	R	0	hypothetical protein/tetrapyrrole methylase family protein
437	107	*atpA*		COG0056	C	1	ATP synthase subunit A
438	107	*atpG*		COG0224	C	1	ATP synthase subunit C
439	107	*atpD*		COG0055	C	1	ATP synthase subunit B
449	106	*murC*		COG0773	M	1	UDP-N-acetylmuramate--L-alanine ligase
452	106	*tgt*		COG0343	J	0	queuine tRNA-ribosyltransferase
453	106	*smf*		COG0758	LU	0	smf protein
458	106	*gidB*		COG0357	M	0	glucose-inhibited division protein B
460	105	*yggV*	*rdgB*	COG0127	F	1	putative deoxyribonucleotide triphosphate pyrophosphatase
462	105	*purH*		COG0138*	F	1	bifunctional phosphoribosylaminoimidazolecarboxamide formyltransferase/IMP cyclohydrolase
470	105	*atpH*		COG0712	C	1	ATP synthase subunit D
475	104	*purF*		COG0034	F	0	amidophosphoribosyltransferase
496	103	*purE*		COG0041	F	1	phosphoribosylaminoimidazole carboxylase catalytic subunit
497	103	*murF*		COG0770	M	1	UDP-N-acetylmuramoylalanyl-D-glutamyl-2,6-diaminopimelate-D-alanyl-D-alanyl ligase
**FUSED DUPLICATES**							
207	120	*guaB*		COG0516<br>COG0517	F<br>R	0	inositol-5-monophosphate dehydrogenase
233	116	*polA*		COG0258<br>COG0749	L<br>L	0	DNA polymerase I
264	114	*metG*		COG0073<br>COG0143	R<br>J	0	methionyl-tRNA synthetases
**MIXED DUPLICATES**							
328	112	*birA*		COG0340	H	0	biotin--protein ligase
407	108	*secD*		COG0342	U	1	protein export protein SecD
442	107	*topA*		COG0550<br>COG0551	L<br>L	0	DNA topoisomerase I

Nr – cluster number from OrthoMCL; # – number of genes in cluster; COG – category according to COG, where COG groups marked with a star (*) have been classified using COGnitor at NCBI; Cat – COG category, with Jr indicating a ribosomal protein; Op – indicates weak (0) and strong (1) operon genes, where a strong operon gene is organized in an operon in more than 80% of the genomes investigated. The operon data are based on the operon predictions from Janga et al. [1]. Singletons are genes that are found with only one copy in the genome, while duplicates have one or more copies (paralogs). In the fused category over 50% of the genes consist of fused domains; while in the mixed category between 10-50% of the genes in the gene cluster contain fused genes.

Table S3 – Genes represented in our data set that are not identified as essential by Gil et al. [2] or Baba et al. [3]

Gene	COG-category
aroA	E
aroC	E
carA	EF
carB	EF
clpB	O
clpX	O
fabF	IQ
folP	H
gidA	D
gidB	M
guaA	F
guaB	FR
mfd	LK
miaA	J
nusB	K
ppnK	G
priA	L
purA	F
purD	F
purE	F
purF	F
purH	F
purL	F
purM	F
pyrB	F
recA	L
recR	L
rncS	K
rpoD	K
ruvA	L
ruvB	L
sfhB	J
smf	LU
sms	O
tgt	J
thdF	R
tig	O
truA	J
truB	J
uvrA	L
uvrB	L
uvrC	L
uvrD	L
yaeS	I
ycfB	J
ychB	I
yebC	S
yfjA	J
yggJ	S
yggV	F
yggW	H
yhhF	L
yjfH	J

Table S4 – Distribution of singletons, duplicates and ribosomal proteins in strong operon and weak operons in the predictions from Janga et al. [1].

	**Strong operon**	**Weak operon**
**Singletons**	5900	1897
**Duplicates**	7353	2817
**Ribosomal**	4347	738

Table S5 – Counting of identical pairs (singletons-singletons, duplicates-duplicates) vs. mixed pairs (singletons-duplicates, duplicates-singletons) in the predictions by Janga et al. [1] (which consists of so-called "gene pairs"), to test whether operons preferably consist of a single category or a mix of the two categories. To perform this analysis, all the operons were treated individually, and the genes in each operon in each bacterium were checked to see if the gene pair consisted of identical pairs or mixed pairs. The total result over all organisms is presented in the table.

	**Singletons**	**Duplicates**
**Singletons**	2854	2753
**Duplicates**	3332	4916

Table S6 – The table show to which degree the different COG-categories are arranged in operons according to predictions from Janga et al. [1].

	COG functional class	Average operon degree
**C**	Energy production and conversion	0.85
**M**	Cell wall/membrane/envelope biogenesis	0.85
**S**	Function unknown	0.84
**J (incl. r-prot)**	Translation, ribosomal structure and biogenesis	0.82
**I**	Lipid transport and metabolism	0.80
**K**	Transcription	0.80
**D**	Cell cycle control, cell division, chromosome partitioning	0.78
**E**	Amino acid transport and metabolism	0.78
**J (excl. r-prot)**	Translation, ribosomal structure and biogenesis	0.77
**Q**	Secondary metabolites biosynthesis, transport and catabolism	0.77
**R**	General function prediction only	0.77
**F**	Nucleotide transport and metabolism	0.76
**H**	Coenzyme transport and metabolism	0.76
**U**	Intracellular trafficking, secretion and vesicular transport	0.74
**T**	Signal transduction mechanisms	0.71
**G**	Carbohydrate transport and metabolism	0.70
**L**	Replication, recombination and repair	0.70
**O**	Posttranslational modification, protein turnover, chaperones	0.67

Table S7 – The number of shared pathways for the different groups. The numbers shown are total number of shared pathways over all essential gene pairs found in KEGG. The table also shows the average number of shared pathways after 1000 permutations, and estimated p-values for getting a number of shared pathways after permutation that is larger than the observed one. The number of genes of each class in the full data set, found in KEGG and found as shared pathway pairs in KEGG is shown.

Interaction		Ribosomal (45/45/45)	Strong operon (73/44/41)	Weak operon (86/45/43)
**Ribosomal**	KEGG db	990	0	0
	Permutation	-	-	-
	P-value	-	-	-
**Strong operon**	KEGG db		78	175
	Permutation		61	198
	P-value		0.001	0.988
**Weak operon**	KEGG db			152
	Permutation			145
	P-value			0.095

Figure S1 – Visualization of cluster sizes

Figure S2 – Phylogram based on hierarchical clustering (dendrogram) from EDE distances.

Figure S3 – Density plot of number of KEGG pathways for strong and weak operon genes.

Figure S4 – Distinction between orthologs and paralogs

The figure shows how gene paralogs are removed from a cluster C after identification of the most likely true ortholog. The OrthoMCL cluster C contains three genes from the same organism X (genXa, genXb, genXc), i.e. the result of gene duplication. The Blast results for all non-duplicated genes in the same cluster are retrieved, here starting with organism A (genA). The gene copies are assigned rank scores R according to the Blast output file. This is used to compute a total rank score S, which is the sum of rank scores from Blast files for all non-duplicated genes in the cluster (i.e. for organism A, B, C, …). An updated cluster C' is made, where only the gene copy with the lowest total rank score is kept. The largest possible rank score R_{max} for a gene will be equal to the number of gene copies, and this maximum value is assigned to any gene copy that is missing in a given Blast output file (the length of the Blast output was restricted by E-value and total number of hits). The smallest possible total rank score S_{min} is equal to the number of non-duplicated genes in the cluster, i.e. the gene has a rank score of 1 in all Blast runs with genes from that cluster.

References

1. Janga SC, Lamboy WF, Huerta AM, Moreno-Hagelsieb G: **The distinctive signatures of promoter regions and operon junctions across prokaryotes**. *Nucleic Acids Res* 2006, **34**(14):3980-3987.
2. Gil R, Silva FJ, Pereto J, Moya A: **Determination of the core of a minimal bacterial gene set**. *Microbiol Mol Biol Rev* 2004, **68**(3):518-537.
3. Baba T, Ara T, Hasegawa M, Takai Y, Okumura Y, Baba M, Datsenko KA, Tomita M, Wanner BL, Mori H: **Construction of Escherichia coli K-12 in-frame, single-gene knockout mutants: the Keio collection**. *Mol Syst Biol* 2006, **2**:2006 0008.

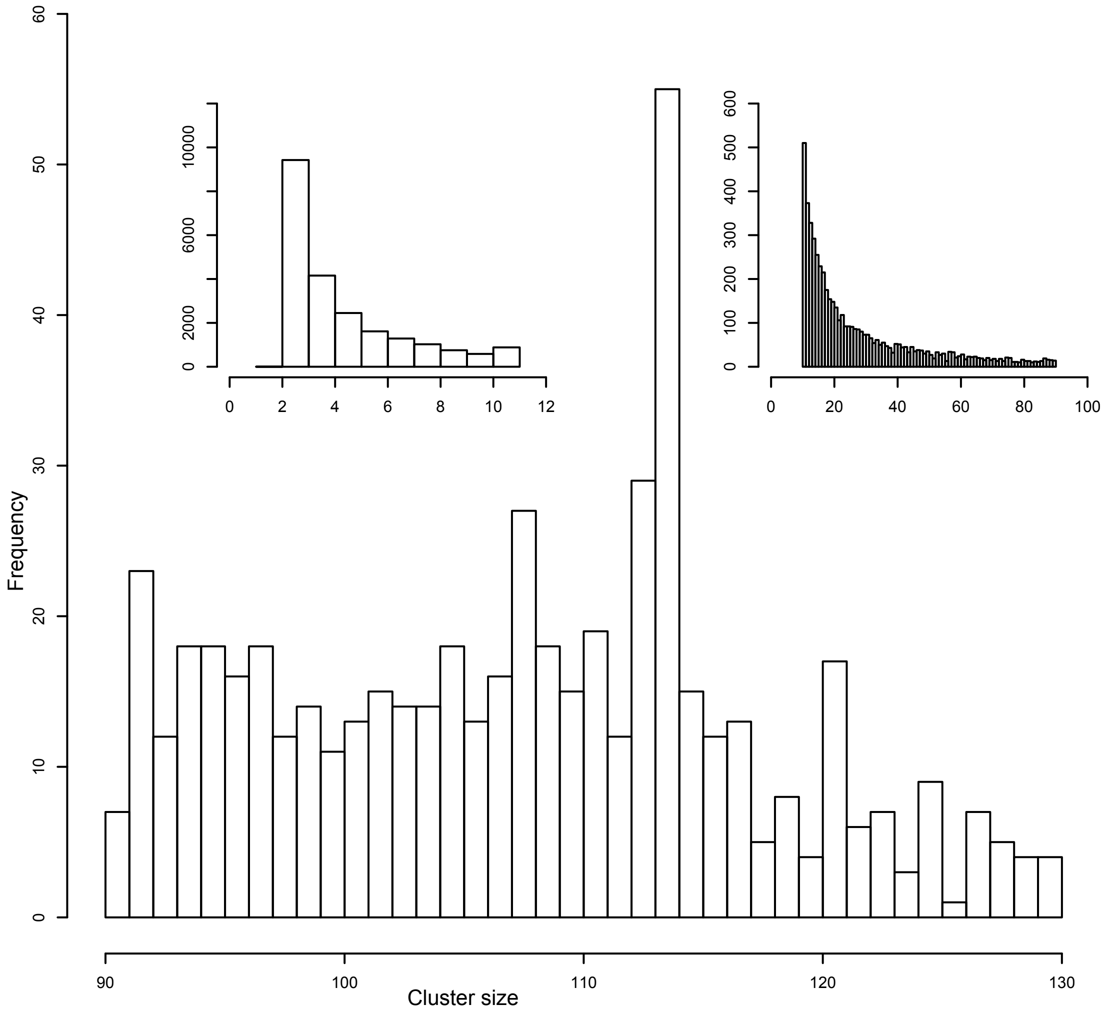
Frequency
Cluster size
0
10
20
30
40
50
60
90
100
110
120
130
0
2000
6000
10000
0
2
4
6
8
10
12
0
100
200
300
400
500
600
0
20
40
60
80
100

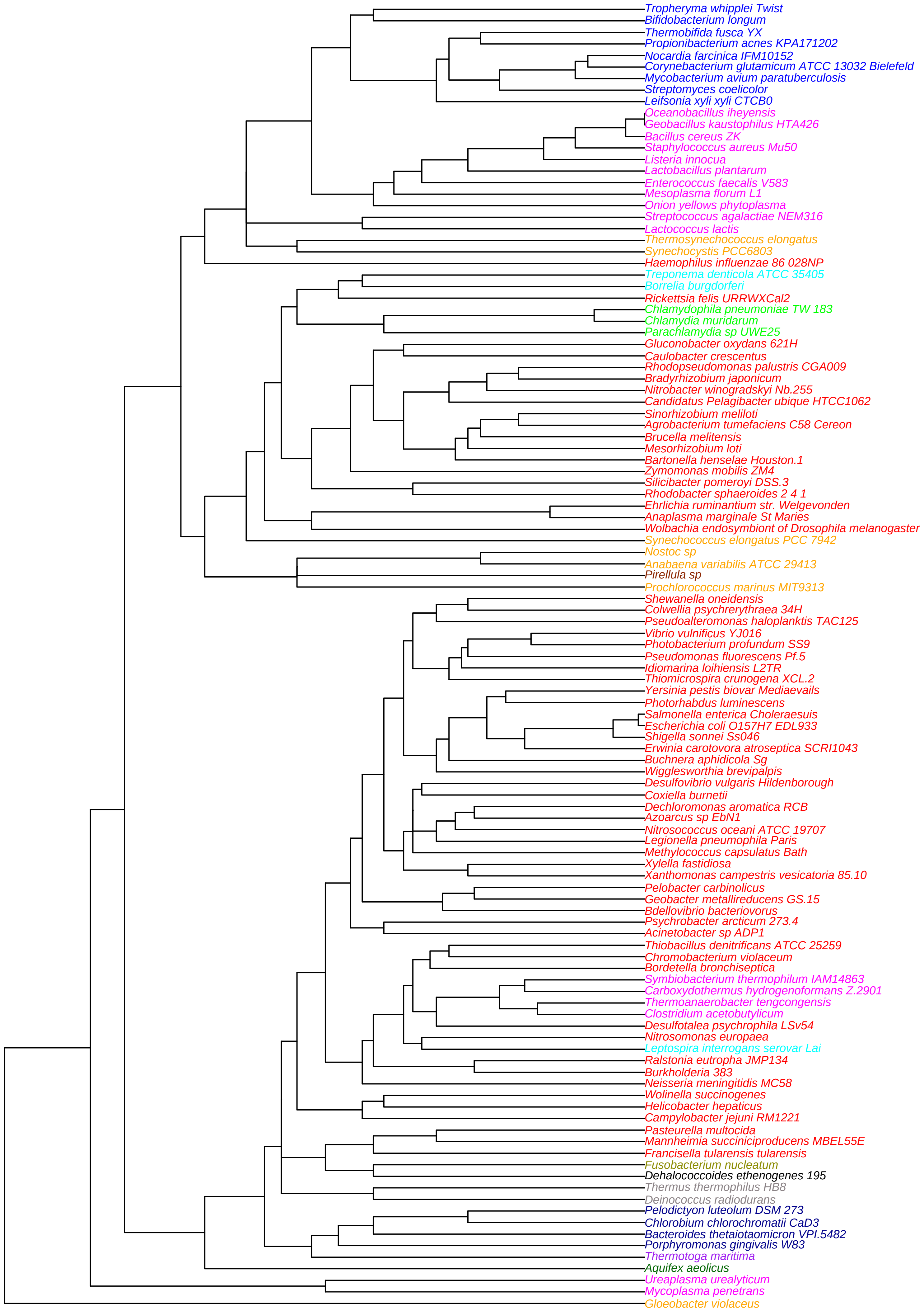
Tropheryma whipplei Twist
Bifidobacterium longum
Thermobifida fusca YX
Propionibacterium acnes KPA171202
Nocardia farcinica IFM10152
Corynebacterium glutamicum ATCC 13032 Bielefeld
Mycobacterium avium paratuberculosis
Streptomyces coelicolor
Leifsonia xyli xyli CTCB0
Oceanobacillus iheyensis
Geobacillus kaustophilus HTA426
Bacillus cereus ZK
Staphylococcus aureus Mu50
Listeria innocua
Lactobacillus plantarum
Enterococcus faecalis V583
Mesoplasma florum L1
Onion yellows phytoplasma
Streptococcus agalactiae NEM316
Lactococcus lactis
Thermosynechococcus elongatus
Synechocystis PCC6803
Haemophilus influenzae 86 028NP
Treponema denticola ATCC 35405
Borrelia burgdorferi
Rickettsia felis URRWXCal2
Chlamydophila pneumoniae TW 183
Chlamydia muridarum
Parachlamydia sp UWE25
Gluconobacter oxydans 621H
Caulobacter crescentus
Rhodopseudomonas palustris CGA009
Bradyrhizobium japonicum
Nitrobacter winogradskyi Nb.255
Candidatus Pelagibacter ubique HTCC1062
Sinorhizobium meliloti
Agrobacterium tumefaciens C58 Cereon
Brucella melitensis
Mesorhizobium loti
Bartonella henselae Houston.1
Zymomonas mobilis ZM4
Silicibacter pomeroyi DSS.3
Rhodobacter sphaeroides 2 4 1
Ehrlichia ruminantium str. Welgevonden
Anaplasma marginale St Maries
Wolbachia endosymbiont of Drosophila melanogaster
Synechococcus elongatus PCC 7942
Nostoc sp
Anabaena variabilis ATCC 29413
Pirellula sp
Prochlorococcus marinus MIT9313
Shewanella oneidensis
Colwellia psychrerythraea 34H
Pseudoalteromonas haloplanktis TAC125
Vibrio vulnificus YJ016
Photobacterium profundum SS9
Pseudomonas fluorescens Pf.5
Idiomarina loihiensis L2TR
Thiomicrospira crunogena XCL.2
Yersinia pestis biovar Mediaevails
Photorhabdus luminescens
Salmonella enterica Choleraesuis
Escherichia coli O157H7 EDL933
Shigella sonnei Ss046
Erwinia carotovora atroseptica SCRI1043
Buchnera aphidicola Sg
Wigglesworthia brevipalpis
Desulfovibrio vulgaris Hildenborough
Coxiella burnetii
Dechloromonas aromatica RCB
Azoarcus sp EbN1
Nitrosococcus oceani ATCC 19707
Legionella pneumophila Paris
Methylococcus capsulatus Bath
Xylella fastidiosa
Xanthomonas campestris vesicatoria 85.10
Pelobacter carbinolicus
Geobacter metallireducens GS.15
Bdellovibrio bacteriovorus
Psychrobacter arcticum 273.4
Acinetobacter sp ADP1
Thiobacillus denitrificans ATCC 25259
Chromobacterium violaceum
Bordetella bronchiseptica
Symbiobacterium thermophilum IAM14863
Carboxydothermus hydrogenoformans Z.2901
Thermoanaerobacter tengcongensis
Clostridium acetobutylicum
Desulfotalea psychrophila LSv54
Nitrosomonas europaea
Leptospira interrogans serovar Lai
Ralstonia eutropha JMP134
Burkholderia 383
Neisseria meningitidis MC58
Wolinella succinogenes
Helicobacter hepaticus
Campylobacter jejuni RM1221
Pasteurella multocida
Mannheimia succiniciproducens MBEL55E
Francisella tularensis tularensis
Fusobacterium nucleatum
Dehalococcoides ethenogenes 195
Thermus thermophilus HB8
Deinococcus radiodurans
Pelodictyon luteolum DSM 273
Chlorobium chlorochromatii CaD3
Bacteroides thetaiotaomicron VPI.5482
Porphyromonas gingivalis W83
Thermotoga maritima
Aquifex aeolicus
Ureaplasma urealyticum
Mycoplasma penetrans
Gloeobacter violaceus

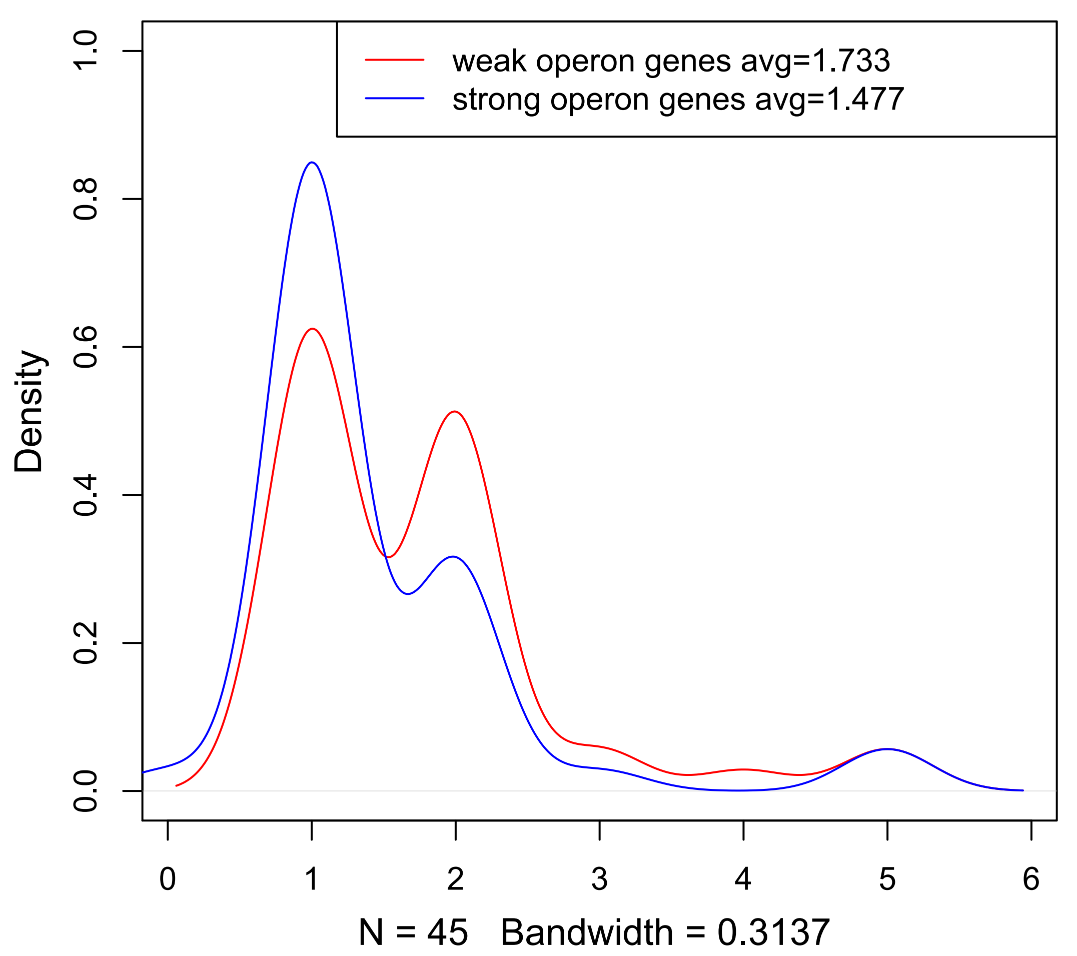
weak operon genes avg=1.733
strong operon genes avg=1.477
Density
0.0
0.2
0.4
0.6
0.8
1.0
0
1
2
3
4
5
6
N = 45   Bandwidth = 0.3137

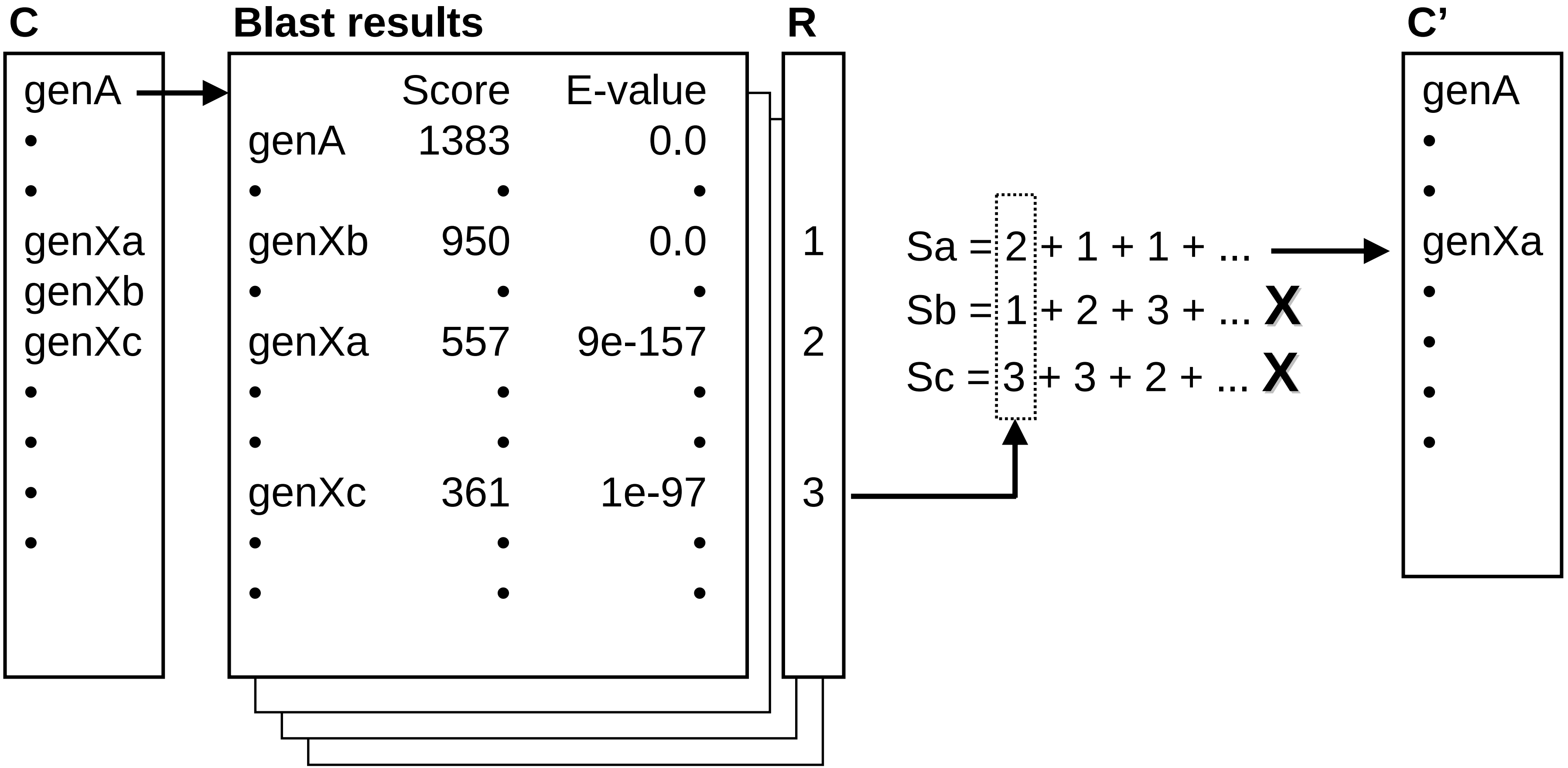
C
Blast results
R
C’
genA
genXa
genXb
genXc
Score
E-value
genA 1383 0.0
genXb 950 0.0
genXa 557 9e-157
genXc 361 1e-97
1
2
3
Sa = 2 + 1 + 1 + ...
Sb = 1 + 2 + 3 + ... X
Sc = 3 + 3 + 2 + ... X
genA
genXa
